# Homoplasy in genome-wide analysis of rare amino acid replacements: the molecular-evolutionary basis for Vavilov's law of homologous series

**DOI:** 10.1186/1745-6150-3-7

**Published:** 2008-03-17

**Authors:** Igor B Rogozin, Karen Thomson, Miklós Csürös, Liran Carmel, Eugene V Koonin

**Affiliations:** 1 National Center for Biotechnology Information, National Library of Medicine, National Institutes of Health, Bethesda, MD 20894, USA; 2 Department of Computer Science, University of New Orleans, New Orleans, LA 70148, USA; 3 Department of Computer Science and Operations Research, Université de Montréal, Montréal, Québec H3C 3J7, Canada

## Abstract

**Background:**

Rare genomic changes (RGCs) that are thought to comprise derived shared characters of individual clades are becoming an increasingly important class of markers in genome-wide phylogenetic studies. Recently, we proposed a new type of RGCs designated RGC_CAMs (after Conserved Amino acids-Multiple substitutions) that were inferred using genome-wide identification of amino acid replacements that were: i) located in unambiguously aligned regions of orthologous genes, ii) shared by two or more taxa in positions that contain a different, conserved amino acid in a much broader range of taxa, and iii) require two or three nucleotide substitutions. When applied to animal phylogeny, the RGC_CAM approach supported the coelomate clade that unites deuterostomes with arthropods as opposed to the ecdysozoan (molting animals) clade. However, a non-negligible level of homoplasy was detected.

**Results:**

We provide a direct estimate of the level of homoplasy caused by parallel changes and reversals among the RGC_CAMs using 462 alignments of orthologous genes from 19 eukaryotic species. It is shown that the impact of parallel changes and reversals on the results of phylogenetic inference using RGC_CAMs cannot explain the observed support for the Coelomata clade. In contrast, the evidence in support of the Ecdysozoa clade, in large part, can be attributed to parallel changes. It is demonstrated that parallel changes are significantly more common in internal branches of different subtrees that are separated from the respective common ancestor by relatively short times than in terminal branches separated by longer time intervals. A similar but much weaker trend was detected for reversals. The observed evolutionary trend of parallel changes is explained in terms of the covarion model of molecular evolution. As the overlap between the covarion sets in orthologous genes from different lineages decreases with time after divergence, the likelihood of parallel changes decreases as well.

**Conclusion:**

The level of homoplasy observed here appears to be low enough to justify the utility of RGC_CAMs and other types of RGCs for resolution of hard problems in phylogeny. Parallel changes, one of the major classes of events leading to homoplasy, occur much more often in relatively recently diverged lineages than in those separated from their last common ancestor by longer time intervals of time. This pattern seems to provide the molecular-evolutionary underpinning of Vavilov's law of homologous series and is readily interpreted within the framework of the covarion model of molecular evolution.

**Reviewers:**

This article was reviewed by Alex Kondrashov, Nicolas Galtier, and Maximilian Telford and Robert Lanfear (nominated by Laurence Hurst).

## Background

With the rapid growth of the collection of sequenced genomes from diverse taxa, reconstruction of the evolutionary history of organisms on the basis of full-scale comparison of their genomes becomes feasible and often seems to be the strategy of choice for phylogenetic analysis [1,2]. Rare genomic changes (RGCs) that are unique to specific clades are often viewed as particularly promising phylogenetic markers [3-6]. The RGCs are shared derived characters (synapomorphies or 'Hennigian' markers that form the basis of classical cladistics [3,6,7]) manifest at the genomic level. By definition, RGCs are chosen such that there is a reasonable degree of confidence that each of them is caused by a single (or a few) rare mutational event. Some of the most prominently used RGCs include retroposon integrations, insertions and deletions (indels) of introns and large protein segments, evolutionary conserved motifs in proteins, protein domain fusions, changes in gene order, and genetic code variants [3,4,8,9]. In many recent studies, RGCs have been employed to infer phylogenetic trees, typically, by using maximum parsimony [3,4,6,10]. The emerging consensus seems to be that RGCs often are phylogenetically informative. In cases where sequence data generate conflicting or equivocal results, RGCs offer an independent way of evaluating alternative phylogenies. As a case in point, RGC analysis has recently suggested substantial revisions of the deep branching of evolutionary trees for eukaryotes [11,12] and prokaryotes [13]. However, systematic identification of genomic changes that qualify as RGCs but are sufficiently numerous to be employed for reliable phylogenetic analysis remains a major challenge [3].

Recently, we proposed a new class of RGCs designated RGC_CAMs (after Conserved Amino acids-Multiple substitutions), which are inferred using a genome-scale analysis of protein and underlying nucleotide sequence alignments [14]. The RGC_CAM approach involves analysis of amino acid residues that are conserved in the major lineages within an analyzed taxonomic division (e.g., eukaryotes), with the exception of a few species that possess a different residue which could be a shared derived character of the corresponding clade. The RGC_CAM analysis has been combined with several statistical tests of competing phylogenetic hypotheses and has been shown to be robust to branch length differences and taxon sampling within a broad range of variation [14].

The RGC_CAM analysis was performed under the assumption that any character shared by the included major eukaryotic lineages (plants, animals, fungi, and Apicomplexa) is the ancestral state whereas the deviating species possess a derived state. The principal obstacle to any RGC methods is homoplasy, i.e., the same amino acid replacements that occur in different lineages independently and thus do not reflect common ancestry but rather represent parallel, reverse, or convergent changes [15]. The RGC_CAMs were specifically designed to mimimize the level of homoplasy by analyzing only those amino acid replacements that require two or three nucleotide substitutions. Multiple substitutions are rare, so the chance to encounter homoplasy is expected to be much lower compared to amino acid changes that require single nucleotide substitutions [16,17], making these replacements plausible RGCs.

The RGC_CAM method is not homoplasy-free [14]. Here we present a scheme to directly estimate the numbers of events leading to homoplasy, namely, parallel changes and reversals. We show that taking homoplasy into account only reinforced the phylogenetic conclusions of the RGC_CAM analysis. In addition, it is demonstrated that parallel changes are significantly more common in internal branches of different subtrees that are separated from the common ancestor by relatively short times than in terminal branches separated by longer time intervals. This finding seems to provide the molecular-evolutionary underpinning of Vavilov's law of homologous series and is readily explained within the framework of the covarion model of molecular evolution.

## Results

### Animal phylogeny under the RGC_CAM approach

**Figure 1:**
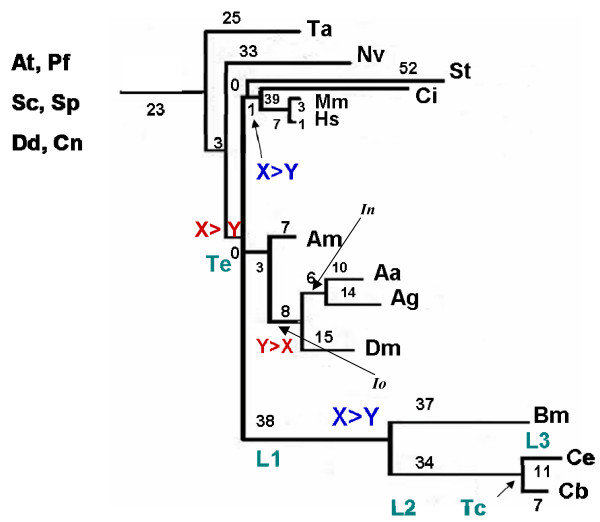
**Animal phylogeny adopted in this study.** The numbers at branches indicated the number of RGC_CAMs which is the measure of branch length. L1 and L2 are internal branch lengths. L3 is the terminal branch length. Tc is the known divergence time for two closely related species (calibration time for L3). Te is the estimated time of the worms-insects-vertebrates divergence. Reversals are shown in red and parallel changes are shown in blue. *Io *indicates the insect internal "old" branch and *In *indicates the insect internal "new" branch (see text). Species abbreviations: *Homo sapiens *(Hs), *Caenorhabditis elegans*(Ce), *Drosophila melanogaster *(Dm), *Saccharomyces cerevisiae *(Sc), *Schizosaccharomyces pombe *(Sp), *Arabidopsis thaliana *(At), *Anopheles gambiae *(Ag), *Plasmodium falciparum *(Pf), *Caenorhabditis briggsae *(Cb), and *Mus musculus *(Mm), *Brugia malayi *(Bm), *Aedes aegypti *(Aa), *Ciona intestinalis *(Ci), *Apis mellifera *(Am), *Cryptococcus neoformans *(Cn), *Dictyostelium discoideum *(Dd), *Nematostella vectensis *(Nv), *Strongylocentrotus purpuratus *(St), and *Trichoplax adhaerens *(Ta)

The animal phylogeny adopted in this study is shown in Figure 1. The branch lengths were estimated in RGC_CAM units. In agreement with previous findings [18], it was found that the three available nematode gene sets comprise a fast-evolving lineage (Figure 1) which would often lead to errors when conventional phylogenetic methods are applied [5,19,20]. Mammals represent the most slowly evolving clade but the deuterostome clade shows high variability of evolutionary rates owing to the fast evolving sea urchin and Ciona lineages (Figure 1). Insects have slower evolutionary rates compared to nematodes, and the insect and nematode clades show relatively little variation of evolutionary rates (Figure 1).

As shown previously, the RGC_CAM approach supported the coelomate clade that unites deuterostomes with arthropods as opposed to the ecdysozoan (molting animals) clade that encompasses arthropods and nematodes [14,21]. This conclusion is compatible with the results of some of the previous genome-wide phylogenetic analyses [22-26] whereas other such analyses claim support for ecdysozoa [27-29]. Moreover, the ecdysozoan topology is currently favored in the evo-devo community, on the basis of the apparently all-important shared feature, namely, molting [30,31]. Interestingly, a subsequent re-analysis of RGC_CAMs, with the sequences from the sea anemone *Nematostella vectensis *included in the set of outgroup species, claimed support for Ecdysozoa [32].

**Figure 2:**
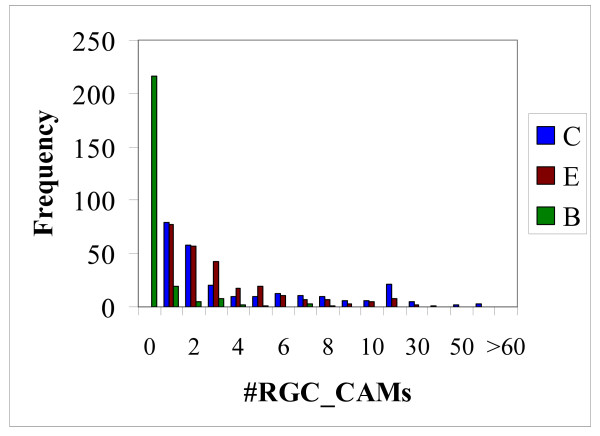
Distribution of the numbers of RGC_CAMs supporting each of the three compared phylogenetic hypotheses in 255 sampling experiments; C = Coelomata, E = Ecdysozoa, and B = 'bizarre' topology (grouping of deuterosomes with nematodes to the exclusion of insects).

We further explored the support for different topologies from RGC_CAM analysis by performing taxon sampling on the outgroup species set. All combinations of 12 to 19 species, i.e., including from one to 8 outgroup species (255 samples altogether), were analyzed (see Additional File 1). For 85 combinations of species, the raw number of RGC_CAMs compatible with the coelomate topology was greater than the number of RGC_CAMs compatible with the ecdysozoa topology, whereas the reverse was true of 88 combinations, with the remaining 82 combinations showing the same number of RGC_CAMs for both topologies (Additional file 1). The number of RGC_CAMs in favor of the "bizarre" topology (grouping of mammals with nematodes to the exclusion of insects) was markedly smaller (Figure 2 and Additional file 1). Thus, when raw numbers are considered, the levels of support for the coelomate topology and the ecdysozoan topology are nearly the same. However, as shown previously, when the branch lengths are taken into account, the support for the coelomate clade becomes substantially greater than that for ecdysozoa [21].

Nevertheless, the two conflicting signals remain. The simplest explanation for this conflict is homoplasy, and indeed, it has been shown that, although the RGC_CAM approach is designed to minimize this effect, it is not homoplasy-free [14,21]. In the following two sections, we directly assess the level of homoplasy among RGC_CAMs.

### Homoplasy: parallel changes

**Figure 3:**
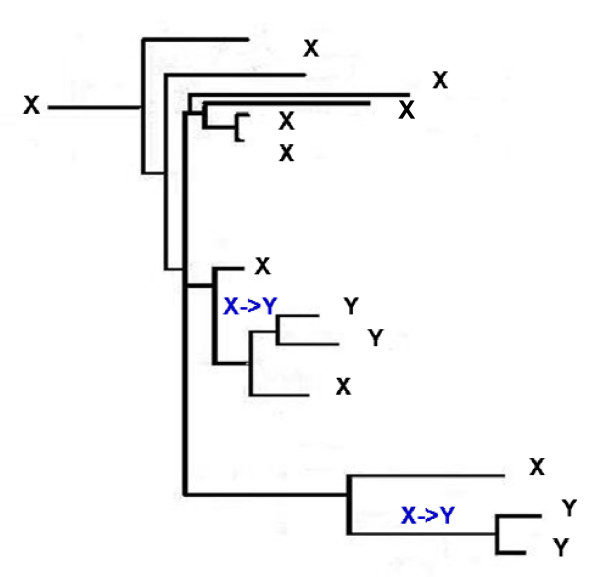
**Identification of parallel changes X->Y.** The tree is the same as in Figure 1 except that the some of the outgroups were collapsed and species names are not indicated for simplicity.

**Figure 4:**
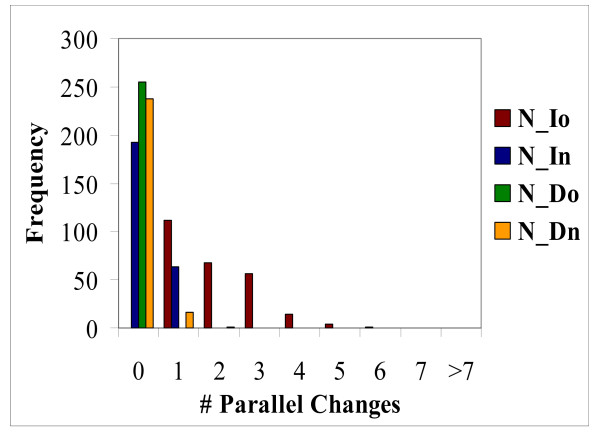
**Distribution of parallel changes between nematodes and deuterostomes in 255 sampling experiments.** The branches of the tree are designated: *N*, nematodes, *Io*, Insect "old", *In*, Insect "new", *Do*, deuterostome "old", *Dn*, Deuterostome "new" (see text for details).

**Figure 5:**
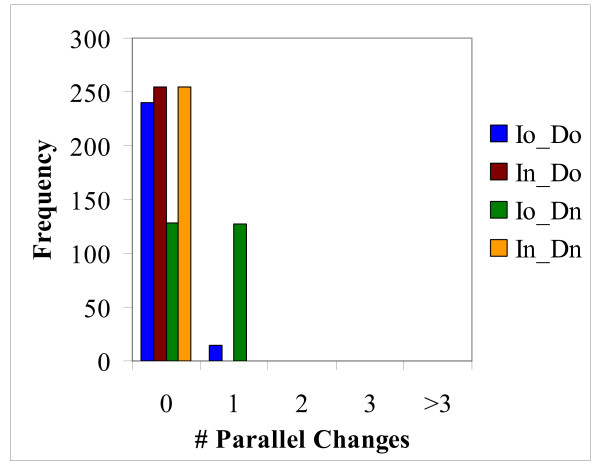
**Distribution of parallel changes between insects and deuterostomes in 255 sampling experiments.** The designations of the tree branches are the same as in Figure 4.

There are two types of evolutionary events that would lead to homoplasy in the RGC_CAM analysis, namely, parallel changes and reversals (Figure 1) [14,21,32]. The RGC_CAM approach provides for the possibility to estimate the level of homoplasy directly. To obtain an estimate of the number of parallel changes, we employed the scheme shown in Figure 3. We required that the same amino acid is shared by two or three pairs of closely related species (e.g. two mosquitoes and two *Caenorhabditis *species) (Figure 3) under the condition that the pair (or triple) of species must have a closely related outgroup which contains the ancestral amino acid. In this case, two parallel changes is the most parsimonious explanation for the observed pattern because all other scenarios require at least three events (Figure 3). All combinations of 12 to 19 species, i.e., including from one to 8 outgroup species (255 samples altogether), were analyzed (Additional File 1). For both insects and deuterostomes, we analyzed two internal branches, one of which was closer to the root ("old") and the other one was closer to the leaves ("new") (Figure 1). Parallel changes in these branches were identified by performing taxon sampling of the outgroup species (Additional File 1). Taxon sampling was necessary because both the number of RGC_CAMs supporting a given topology and the number of observed parallel changes critically depend on the composition of the outgroup. When all 8 or any combination of 7 outgroup species are included, there are no RGC_CAMs in the deuterostome branch, the insect branch becomes very short as well, and only one or two parallel changes are seen (Additional File 1). In contrast, for many combinations of smaller numbers of outgroup species, several parallel changes between nematodes (*N*) and insects, particularly, in the "old" internal branches (*Io*) were detected (Figure 4 and Additional File 1). The insect "old" branch *Io *is only slightly longer than the "new" branch *In *(8 RGC_CAMs and 6 RGC_CAMs, respectively; Figure 1), so the substantial excess of parallel changes in the "old" branch was unexpected. The same pattern was seen between the "old" and "new" insect branches and the "new" deuterostome branch, with a much greater number of parallel changes occurring in the "old" insect branch (Figure 5 and Additional File 1). Thus, the rate of parallel changes is not uniform, with deeper, more ancient branches being more prone to parallel changes.

We further employed a relaxed scheme for parallel change detection where these changes were detected between terminal branches (species) rather than between internal branches as in the analyses described above. Specifically, the branches leading to the two *Caenorhabditis *species and those leading to the two mosquito species were compared. The *Caenorhabditis *terminal branches are approximately three times shorter than the internal nematode branch, whereas the terminal mosquito branches are approximately twice as long as the "new" internal insect branch (Figure 1). Accordingly, one would expect to observe a number of parallel changes close to that in the *N*_*In *comparison (Figure 4). However, we detected no parallel changes in any of the 4 comparisons. This result is unlikely to be due to short branch lengths because parallel changes were readily detected for much shorter "old" internal branches, e.g., in the *Io*_*Do *comparison (Figure 5). The absence of parallel changes in the terminal branches is consistent with the excess of parallel changes in deep branches of phylogenetic trees described above.

To determine the statistical significance of this trend, we used the following simplified scheme. The number of unique parallel changes in selected branch pairs was tallied from the 255 sampling experiments (all repeated parallel changes were removed from this analysis, thus the resulting number was the union of parallel changes detected in individual experiments). Specifically, the *N*_*Io*, *N*_*In*, *N*_Ag, and *N*_Aa comparisons were analyzed. For *N*_*Io*, 6 unique parallel changes were detected, whereas the other three comparisons taken together yielded 3 unique parallel changes (Table 1). The internal nematode branch *N *is the same for all comparisons, thus the frequency of parallel changes depends on the length of the insect branches only. The ratio of the length of the *Io *branch to the total length of all involved branches is ~0.2 (Table 1). The probability to observe 6 of the total of 9 detected parallel changes in this relatively short branch is 0.003 under the binomial test.

**Table 1:** Comparison of parallel changes in internal and terminal branches of nematodes, insects and deuterostomes

	Branches where parallel changes were analyzed			
	
	*N*_*Io*	*N*_*In*	*N*_Ag	*N*_Aa
#RGC_CAMs (%)	6 (67)	1 (11)	0 (0)	2 (22)
Relative branch length	0.201	0.169	0.365	0.265
Normalized #parallel changes	29.9	5.9	0.	7.5

#RGC_CAs (%)	45 (45)	22 (22)	15 (15)	19 (19)
Relative branch length	0.205	0.205	0.340	0.250
Normalized #parallel changes	219.5	107.3	44.1	76.

	*N*_*Do*	*N*_*Dn*	*N*_Hs	*N*_Mm

#RGC_CAMs (%)	0 (0)	2 (100)	0 (0)	0 (0)
Relative branch length	0.094	0.621	0.109	0.176
Normalized #parallel changes	0.	3.2	0.	0.

#RGC_CAs (%)	28 (33)	51 (60)	3 (3.5)	3 (3.5)
Relative branch length	0.121	0.581	0.139	0.159
Normalized #parallel changes	231.4	82.1	21.6	18.9

	*Io*_*Do*	*Io_Dn*	*Io*_Hs	*Io_*Mm

#RGC_CAMs (%)	1 (50)	1 (50)	0 (0)	0 (0)
Relative branch length	0.094	0.621	0.109	0.176
Normalized #parallel changes	10.6	1.6	0	0

#RGC_CAs (%)	21 (45)	24 (51)	1 (2)	1 (2)
Branch length	0.121	0.581	0.139	0.159
Normalized #parallel changes	173.6	41.3	7.2	6.3

	*In*_*Do*	*In*_*Dn*	*In*_Hs	*In*_Mm

#RGC_CAMs (%)	0 (0)	0 (0)	0 (0)	0 (0)
Relative branch length	0.094	0.621	0.109	0.176

#RGC_CAs (%)	5 (23)	16 (72)	0 (0)	1 (5)
Relative branch length	0.121	0.581	0.139	0.159
Normalized #parallel changes	41.3	27.5	0.	6.3

*N *is the internal nematode branch, *Io *is the "old" internal insect branch, *In *is the "new" internal insect branch, *Do *is the "old" internal deutrostome branch, *Dn *is the "new" internal deutrostome branch, Ag indicates the terminal branch leading to *Anopheles gambiae*, Aa indicates the terminal branch leading to *Aedes aegypti*, Hs indicates the terminal branch leading to human, Mm indicates the terminal branch leading to mouse. The relative branch length was calculated as the ratio of the length of the analyzed branch to the total length of all involved branches averaged over 255 sampling experiments. Normalized number of parallel changes is the number of parallel changes divided by the branch length.

Although the observed excess of parallel changes in internal branches gained unequivocal statistical support, the raw numbers of parallel changes were small. To increase the resolution of the analysis of parallel changes, we relaxed the definition of RGC by allowing all possible amino acid replacements (as opposed to only those that require two or three nucleotide substations in RGC_CAMs). We denote these characters RGC_CAs. Such relaxation of the original RGC definition is expected to result in a dramatic increase of homoplasy. Thus, much larger raw numbers could be obtained for the analysis of parallel changes although alternative explanations involving combinations of more than two parallel changes and/or reversals simultaneously become more likely. Although numerically the excess of parallel changes in deep branches was less dramatic than in the RGC_CAM comparison, the statistical support for this trend was even stronger (P < 10^-7^) owing to the large number of observations (Table 1). The same trend was found for the other, shorter internal branches, such as *D*_*o *_and *D*_*n *_when parallel changes were measured in RGC_CAs (in this case, the lengths of the branches differed greatly, so the effect was not obvious from the comparison of the raw numbers but became apparent after normalization over branch length; Table 1).

**Figure 6:**
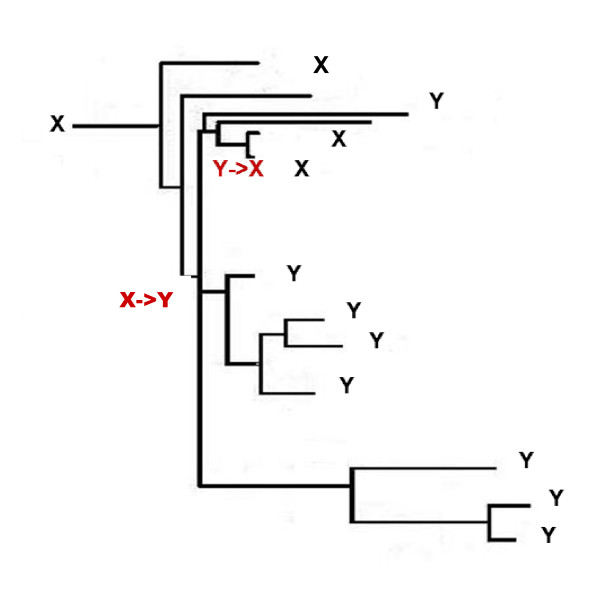
**Identification of reversals X->Y and Y->X.** The tree is the same as in Figure 1 except that the some of the outgroups were collapsed and species names are not indicated for simplicity.

The estimates of the parallel changes presented here could not be directly factored into the RGC_CAM analysis of the Coelomate-Ecdysozoan problem because the informative RGC_CAMs for addressing this problem and the events leading to homoplasy were located on different branches of the phylogenetic tree (Figures 1, 3, and 6). Extrapolation of the estimated parallel changes for the branches leading to the analyzed trifurcation is complicated by the observed increase in the number of parallel changes from the leaves of the tree toward the analyzed trifurcation. Nevertheless, we attempted a crude estimation using the simplest, linear model of the increase in parallel changes depending on the depth of the tree. Specifically, the number of parallel changes between the branches leading from the trifurcation to insects and nematodes was estimated as:

*P*_*e *_= *P*_*N_Io*_(*N*_*t*_/*N*) (*I*_*t*_*/I*_*o*_)*C*^2^

where *Pe *is the estimated number of parallel changes, *P*_*N_Io *_is the observed number of parallel changes between the insect "old" branch and the internal nematode branch, (*N*_*t*_/*N*) is the ratio of the lengths of nematodes-to-trifurcation branch and the internal nematode branch, (*I*_*t*_/*I*_*o*_) is the analogous ratio for insects, and C is the coefficient of linear increase in the number of parallel changes from the leaves of the tree toward the analyzed trifurcation and calculated as the ratio of the numbers of parallel changes between the internal nematode branch and the "old" and "new" insect branches, i.e., *C *= *P*_*N_Io*_/*P*_*N_In*_. In order to obtain more reliable estimates, the *N*_*Io *vs. *N*_*In *were taken from the RGC_CA data (Table 1): C = 45/22~2. The *P*_*e *_values were obtained for each of the 255 sampling experiments (Additional file 1) and were compared to the number of characters supporting Ecdysozoa (Figure 2). The two series of values have close mean values (2.96 for *P*_*e *_and 3.46 for the number of RGC_CAMs supporting ecdysozoa) although the difference is statistically significant (P = 0.02) by Student's *t*-test). Although these estimates are based on simplistic assumptions and should be interpreted with extreme caution, they suggest that the non-negligible support of the Ecdysozoa clade could be, largely, explained by parallel changes, owing, primarily, to the long nematode branch. Analogous estimates for the insect-deuterostome comparison yielded extremely small numbers (0 in most of the sampling experiments) owing to the short deuterostome branch (data not shown) compared to the number of RGC_CAMs supporting the coelomate clade (Figure 2). Thus, parallel changes hardly can explain a substantial fraction of the RGC_CAMs supporting the coelomate clade.

### Homoplasy: reversals

Reversals comprise the second potential source of homoplasy (Figure 1). To obtain an estimate of the number of reversals, we employed the scheme shown in Figure 6. We required the same amino acid to be shared by a pair of closely related species (e.g. human-mouse) and the outgroup species (fungi, plant, protist, *Nematostella vectensis*, and *Trichoplax adhaerens*) but not the rest of the animals (Figure 6). In this case, a reversal in an internal branch is the most parsimonious scenario, assuming that the tree topology in the node leading to vertebrates, insects and nematodes is a true trifurcation. If this is not the case, two parallel changes also might explain the observed pattern for the "old" branches, one in the internal branch leading to the Coelomata (or Ecdysozoa) clade and the other in a terminal branch on the other side of the tree. Thus, the obtained estimates comprise the upper bound of the number of reversals in the case of the "old" internal branches (including the nematode internal branch). For a "new" internal branch, a reversal is the unequivocal most parsimonious scenario. All combinations of 12 to 19 species, i.e., including from one to eight outgroup species (255 samples altogether), were analyzed (Additional file 1).

**Figure 7:**
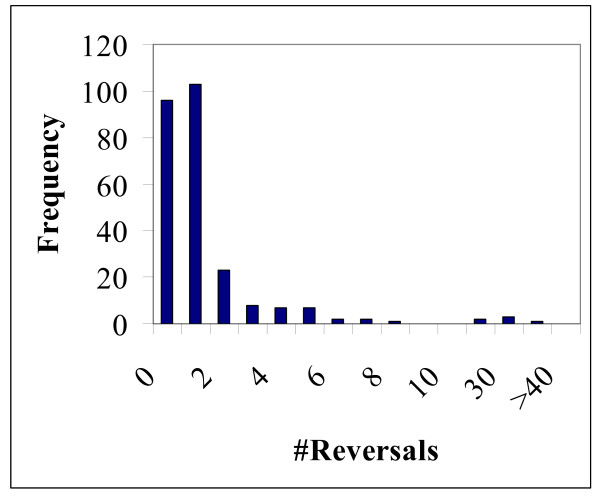
Distribution of reversals on the internal nematode branch in 255 sampling experiments.

A substantial number of reversals were detected in the internal nematode branch (Figure 7) although the number of reversals was significantly (Student's *t*-test, P < 10^-6^) smaller than the number of RGC_CAMs supporting the coelomate clade (Additional File 1). Reversals have been invoked by Irimia et al. [32] to explain (away) the observed RGC_CAM support for the coelomate clade. However, the results presented here along with those in a previous study that was performed on a different set of species [21] show that the number of reversals is insufficient to account for this support.

**Figure 8:**
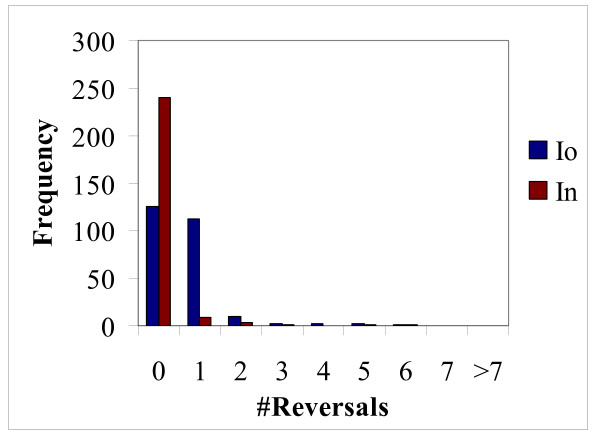
**Distribution of reversals on the internal insect branches in 255 sampling experiments.** The branch designations are as in Figure 4.

**Figure 9:**
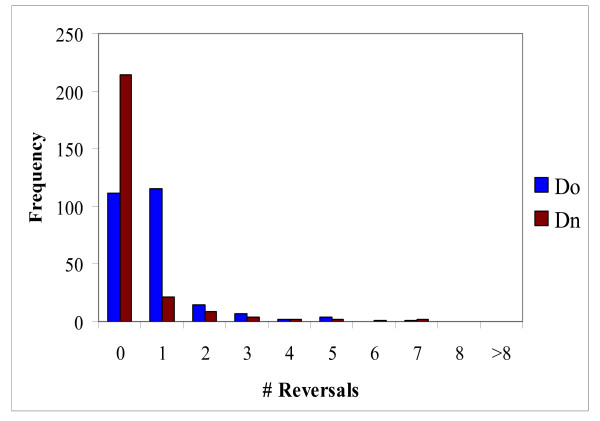
**Distribution of reversals on the internal Deuterostome branches in 255 sampling experiments.** The branch designations are as in Figure 4.

Analysis of reversals in insects and deuterostomes revealed a pattern similar to that observed for parallel changes, i.e., the total number of reversals in the "old" internal branch was greater than the number of reversals in the "new" branch (Figures 1, 8 and 9). However, the difference was small and, in the case of insects, potentially could be attributed to the length difference between the "old" and "new" branches (Figure 1). To test the hypothesis that reversals are more prevalent in deep branches, we employed a scheme where only one *Caenorhabditis *species or one mosquito species shared an RGC_CAM with the outgroup species and other animals. This resulted in a smaller number of probable reversals compared to the internal branches (results not shown), however the pattern was not as obvious as that with parallel changes where none were seen in the terminal branches (see above).

To determine the statistical significance of the apparent excess of reversal in deep branches, we used the same approach as described above for parallel changes. The number of unique reversals in selected branches was calculated from the 255 sampling experiments (all repeated reversals were removed from this analysis, thus the resulting number was the union of the reversals detected in individual experiments) (Table 2). For this test, we chose the insect clade because the bizarre hypothesis never gained any substantial support in any experimental settings (Figure 2 and [22-29]) and is generally not considered a plausible evolutionary scenario [30,31]. Thus two parallel changes as an alternative to a reversal can be effectively ruled out. Substantial numbers of reversals were detected in all analyzed insect branches (Table 2). The differences between branches were not significant (results not shown). However, the raw numbers were small (Table 2) which could hamper the statistical analysis. In order to increase the resolution, we again turned to RGC_CAs (see above) which yielded greater raw numbers of reversals (Table 2). In this RGC_CA analysis, a relatively small but statistically significant (P < 10^-9^) excess of reversals in the "old" branch was observed (Table 2). Thus, reversals seem to show the same, albeit much weaker, evolutionary trend as parallel changes (compare the results in Table 1 and 2).

**Table 2:** Analysis of reversals in internal and terminal branches of insects

	*Branches where reversals were analyzed*			
	
	*Io*	*In*	Ag	Aa
Complete sampling				

#RGC_CAMs (%)	16 (31)	14 (27)	14 (27)	8 (15)
Branch length	0.201	0.169	0.365	0.265
Normalized #reversals	79.6	82.8	38.4	30.2

#RGC_CAs (%)	155 (32)	122 (25)	129 (27)	76 (16)
Branch length	0.205	0.205	0.340	0.250
Normalized #reversals	765.1	595.1	379.4	304.

Sampling with at least two outgroup species				

#RGC_CAMs (%)	7 (33)	6 (29)	6 (29)	2 (9)
Branch length	0.210	0.164	0.351	0.275
Normalized #reversals	34.8	35.5	16.4	7.5

#RGC_CAs (%)	86 (35)	58 (24)	64 (26)	35 (15)
Branch length	0.208	0.196	0.356	0.240
Normalized #reversals	413.5	295.9	179.8	145.8

Sampling with at least three outgroup species				

#RGC_CAMs (%)	3 (60)	1 (20)	1 (20)	0 (0)
Branch length	0.208	0.156	0.345	0.281
Normalized #reversals	14.4	6.4	2.9	0.

#RGC_CAs (%)	46 (28)	28 (61)	28 (5)	14 (6)
Branch length	0.212	0.201	0.366	0.221
Normalized #reversals	217.0	139.3	76.5	63.3

*Io *is the "old" internal insect branch, *In *is the "new" internal insect branch, Ag indicates the terminal branch leading to *Anopheles gambiae*, Aa indicates the terminal branch leading to *Aedes aegypti*. The relative branch length was calculated as the ratio of the length of the analyzed branch to the total length of all involved branches averaged over 255 sampling experiments (complete sampling) or from restricted sampling with at least 2 or 3 outgroup species. Normalized number of reversals is the number of reversals divided by the branch length.

For RGC_CAMs, we assumed that the conserved amino acid in the outgroup species represents the ancestral state. However, two parallel changes (Y->X in the branch leading to the outgroup species and in the analyzed internal branch) also could explain the pattern in Figure 6, and this effect might be especially important when the number of outgroup species is small. To assess the effect of such parallel changes, we required that the outgroup set included, at least, 2 or 3 species. The results were not significantly different from those obtained with the unrestricted taxon sampling (Table 2), suggesting that the absence of dramatic differences between the "old" and "new" branches is a reliable result that does not depend on the number of outgroup species.

As discussed above, a substantial number of reversals were detected in the internal nematode branch (Figure 7); in principle, these reversals might explain (part of) the observed RGC_CAM support for the coelomate clade. We applied the adjustment procedure described in the preceding section to the reversals in the nematode branch. As expected, the estimated number of reversals on the trifurcation-nematode branch was greater than the observed number of reversals on the internal nematode branch (Figure 7). However, the difference, in this case, was relatively minor (a ~1.3-fold increase, on average), and estimated number of reversals was still significantly smaller than the number of RGC_CAMs supporting the coelomate clade (the mean number of reversals was 2.07, and the mean number of supporting RGC_CAMs was 5.54, P < 10^-6 ^by Student's *t*-test).

## Discussion

Homoplasy, arguably, is the principal impediment for all (broadly defined) cladistic approaches in phylogenetics. Here, we present direct estimates of two types of events, namely, parallel changes and reversal, that lead to homoplasy. With regard to the potential effects of homoplasy on the resolution of the Coelomata-Ecdysozoa problem, the present estimates should be interpreted with caution because the informative RGC_CAMs relevant for this problem and the events conducive to the direct estimation of homoplasy were located on different branches of the phylogenetic tree (Figures 1, 3, and 6). Nevertheless, with this caveat, the present provide further support to the Coelomate clade that has been observed in our previous analyses using the RGC_CAM approach [14,21]. The rate of reversals was nearly uniform across the tree, and the number of reversals in the nematode branch was significantly smaller than the number of RGC_CAMs supporting the coelomate clade. An analogous estimate for parallel changes was complicated by the observed substantial increase in the number of parallel changes from the leaves of the tree toward the analyzed trifurcation. Nevertheless, a conservative estimate that was obtained using the simplest, linear model of the increase in parallel changes depending on the depth of the tree showed that parallel changes made negligible contribution to the support of the Coelomate clade, given the very short internal branch leading to Deuterostomes. In contrast, the non-negligible support of the Ecdysozoa clade was, largely, explained by parallel changes, owing to the long nematode branch. Of course, interpretation of these results requires caution as with any result obtained with the parsimony principle. We inferred parallel changes and/or reversals under evolutionary scenarios that, according to maximum parsimony, required two substitutions (Figures 3 and 6). We cannot rule out that non-parsimonious scenarios that involve more than two parallel changes and/or reversals might have some impact on the RGC_CAs and RGC_CAMs analyses. However, it is expected that RGC_CAMs (and, to a lesser extent, RGC_CAs) are, largely, refractory to this problem because the probability of three independent rare events is much smaller than two independent rare events.

**Figure 10:**
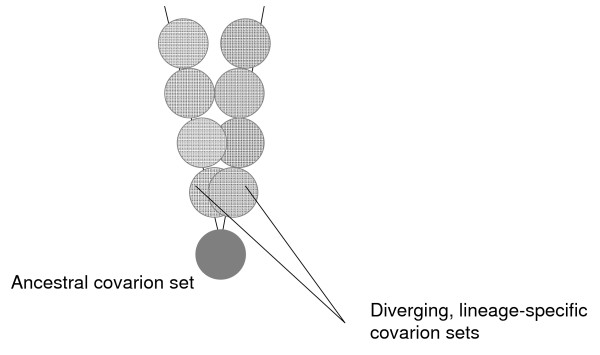
A cartoon representation of the evolution of covarion sets after divergence of two lineages.

Homoplasy is a major problem for phylogenetic methods but it is also an aspect of sequence evolution that deserves analysis in its own right. In particular, the intriguing finding of a dramatic excess of parallel changes in internal branches of a phylogenetic tree compared to terminal branches (leaves) begs an explanation. We believe that such an explanation can be readily found within the framework of the covarion concept of molecular evolution [33-36]. According to the covarion concept, at any given moment in evolution, only a (relatively) small subset of amino acid sites (termed covarions, after concomitantly variable codons) in a protein can accommodate replacements whereas the replacements in the remaining sites are deleterious due to functional constraints. Each fixed replacement changes the set of covarions, rendering replacements in some of the previous covarions inadmissible but simultaneously yielding new covarions. It has been reported that covarion models fit protein sequence comparison data better than simpler models of rate variation in which site constraints remain unchanged throughout time [37,38]. In current mathematical models [37,39-41] that embody the covarion hypothesis, codon sites oscillate between "variable" and "invariable" states independently of each other. Over long evolutionary intervals, the resulting "wave" of covarions encompasses all or most of the protein sequence. Under this model of evolution, two diverging lineages have the same set of covarions immediately after divergence but, with time passing, the overlap between the sets of covarions progressively decreases (Figure 10). This being the case, it becomes obvious that the likelihood of parallel changes drops concomitantly with the decrease of the overlap between the covarion sets of diverging lineages. The possibility of parallel changes is not eliminated altogether because, in the course, of evolution, the same sites can be independently recruited into evolving covarion sets of different lineages.

In a broader evolutionary context, the observed excess of parallel changes seems to provide the molecular-evolutionary underpinning for the "law of homologous series in variation". The law of homologous series was propounded by the famous Russian geneticist, botanist, and plant geographer Nikolai I. Vavilov as a generalization of his extensive observations on the variability of traits in cultured and wild plants [42-46]. In Vavilov's words, "Species and genera that are genetically closely related are characterized by similar series of heritable variations with such regularity that knowing the series of form within the limits of one species, we can predict the occurrence of parallel forms in other species and genera. The more closely related the species...in the general system, the more resemblance will there be in the series of variations." [42]. Owing to its predictive power, the law of homologous series has been touted as the "Mendeleev table of biology" by Vavilov and his disciples [47] but has received relatively little attention in modern research [43]. One obvious reason for this relative neglect would be that the molecular underpinning of Vavilov's law so far has not been elucidated [43]. The excess of parallel changes in deep branches of phylogenetic trees reported here precisely mimics the law of homologous series at the molecular level in that more closely related sequences are more prone to parallel mutations, presumably, because they have substantially overlapping sets of covarions, unlike sequences that have diverged farther (Figure 10). The excess of parallel changes for distantly related species observed in this paper reverberates with the recent finding of a high frequency of parallel changes in evolving protein sequences from mammalian, fruit fly and yeast species [48].

Remarkable as a biological phenomenon, the non-uniform distribution of parallel changes across the tree branches creates a major problem for the RGC_CAM approach as well as for conventional phylogenetic methods because the rate of parallel changes on the informative branches emitting from the analyzed trifurcation is hard to extrapolate. However, as a first approximation, using a simple, linear extrapolation, we showed that, in the specific case of the Coelomata-Ecdysozoa dilemma, taking into account parallel changes does not affect the support for Coelomata but eliminates much of the support for Ecdysozoa.

In contrast, the analysis of reversals under the RGC_CAM approach did not reveal any excess in internal branches compared to terminal branches. When we used a less restrictive approach to character definition in which the requirement of multiple mutations was lifted (RGC_CAs), a weak, although statistically significant trend of the same direction was detected. As with parallel changes, this does not seem surprising under the covarion model because, considering reversals to a particular ancestral state, any site is progressively less likely to remain within the covarion set with increasing time since divergence and therefore the number of reversals (and any other multiple substitutions) could be expected to drop. In practice, however, the estimates of reversals on terminal branches can be used for extrapolation of reversals on the branches emitting from the analyzed trifurcation. Such estimates have shown that the number of reversals is relatively small compared to the number of informative RGC_CAMs.

## Conclusion

Homoplasy is usually considered as a complication in phylogenetics including various flavors of cladistic analysis. The incentive for this study was no exception as we sought to directly estimate the rates of two types of events leading to homoplasy, parallel changes and reversals, in order to assess the effect of homoplasy on the results of the RGC_CAM analysis.

We found that, although a considerable number of parallel changes and reversals were detected, the results of the previous analyses, in particular, the support for the Coelomata clade, as opposed to the Ecdysozoa clade, were not changed by homoplasy. Moreover, parallel changes did not affect the support for Coelomata but accounted for most of the support for Ecdysozoa. In the process of this analysis, we discovered a non-uniform distribution of parallel changes across the branches of phylogenetic trees, with an excess in internal branches as compared to leaves. This non-uniformity creates a potentially serious problem for RGC_CAM and similar analyses because it complicates extrapolation of the number of parallel changes to internal branches. Specifically, however, the support for the Coelomata clade was not compromised. By contrast, most of the observed support for the Ecdysozoa clade could be explained by parallel changes. The excess of parallel changes in deep branches of phylogenetic trees is a notable evolutionary phenomenon in itself. It seems to be readily interpretable in terms of the covarion model of molecular evolution, and provides the molecular-evolutionary underpinning of Vavilov's law of homologous series.

## Materials and methods

### Orthologous protein sets and alignments

Each of the 462 protein alignments analyzed here was constructed from selected euKaryotic Orthologous Groups (KOGs [49]) and included orthologous genes from 10 eukaryotic species with completely sequenced genomes: *Homo sapiens *(Hs), *Caenorhabditis elegans *(Ce), *Caenorhabditis briggsae *(Cb), *Drosophila melanogaster *(Dm), *Saccharomyces cerevisiae *(Sc), *Schizosaccharomyces pombe *(Sp), *Arabidopsis thaliana *(At), *Anopheles gambiae *(Ag), *Plasmodium falciparum *(Pf), and *Mus musculus *(Mm) [14]. Amino acid sequence alignments are available at the authors' Web site [50]. To these KOGs, probable orthologs from several other eukaryotic genomes, namely, those of *Brugia malayi *(Bm), *Aedes aegypti *(Aa), *Ciona intestinalis *(Ci), *Apis mellifera *(Am), *Cryptococcus neoformans *(Cn), *Dictyostelium discoideum *(Dd), *Nematostella vectensis *(Nv), *Strongylocentrotus purpuratus *(St), and *Trichoplax adhaerens *(Ta) were added using the COGNITOR method [51]. Briefly, all the protein sequences from the new genomes are compared to the protein sequences previously included in the KOGs; a protein is assigned to a KOG when two genome-specific best hits to members of the given KOG are detected. To minimize misalignment problems, only conserved, unambiguously aligned regions of the alignments constructed using the MUSCLE program [52] were included in the further analysis. Specifically, all positions containing a deletion or insertion in at least one sequence were removed from the protein sequence alignment together with 5 adjacent positions [14,21].

### Frequency of amino acid substitutions

Biases in amino acid substitutions might represent a substantial problem for phylogenetic analysis [53]. In order to assess the potential effect of such biases on the outcome of the RGC_CAM analysis, we analyzed frequencies of amino acids involved in RGC_CAMs for nematodes, deuterostomes and arthropods (Additional file 2). We found that the distributions of mutated amino acids are not significantly different between these lineages (Additional file 3), and the same result was obtained for the amino acids that result from the RGC_CAM substitutions (Additional files 2 and 3). In addition, we found that the distributions were positively correlated between clades, and for some of the pairwise comparisons, this correlation was statistically significant (Additional files 3). Thus, biases in amino acid substitutions do not appear to present a major problem for the RGC_CAM approach.

### Relaxed molecular clock properties of RGC_CAMs

To assess the validity of RGC_CAMs as phylogenetic characters, we assessed the variation of evolutionary rates measured in RGC_CAM units along the analyzed clades, i.e., the extent of clock-like behavior of RGC_CAMs. For normalization, we turned to molecular dating; considering the controversies in this area [54-56], we employed relatively well established, recent dates of species divergence as calibration points. The following equation was used:

T_e _= T_c _× [(L_1 _+ L_2 _+ L_3_)/L_3_]

where T_e _is the estimated time of the nematodes-insects-vertebrates divergence (this node was assumed to be a trifurcation), T_c _is the divergence time for two closely related species (calibration point), and L_1_, L_2 _and L_3 _are branch lengths, with L_3 _representing the length of a branch emitted by the node corresponding to the given T_c _(Figure 1). This equation is based on the assumption that the rate of RGC_CAM emergence is the same in terminal and internal branches for each clade of animals. Time estimates allow us to test this assumption. We used the following calibration times: 95 Mya for the human-mouse divergence [57], 80 Mya for the *C. elegans *– *C. briggsae *divergence [58], and 250 Mya for *A. gambiae *– *A. aegypti *divergence [59].

The time estimates obtained with the RGC_CAM approach are shown in Additional File 4. There is a substantial variance of the T_e _estimates (348 – 902 Mya) because of the major branch length differences and the small number of RGC_CAMs on some branches (e.g., 3 RGC_CAMs on the mouse branch compared to 1 RGC_CAM on the human branch) which leads to over-dispersion. These time estimates (Additional File 4) are lower than the previously reported early divergence dates for animal phyla, i.e., 970–1040 Mya [57,60]. The mean estimate for the nematodes-insects-vertebrates divergence is 646 Mya. With two branches, *A. gambiae *and *A. aegypti*, lower T_e _values were obtained (Additional File 4); these values are better compatible with more recent divergence dates which, in turn, are consistent with the Cambrian explosion [61,62]. The reasonable consistency of the obtained time estimates (with the exception of two outlier values given by mouse and C. briggsae; see additional File 4) suggests that RGC_CAMs behave as a relaxed molecular clock [63], i.e., the rates of RGC_CAMs emergence are, approximately, the same in the analyzed terminal and internal branches of all three clades. In some cases, length estimates of terminal branches (e.g., the long branch leading to sea urchin) might be unreliable because of population polymorphism and/or sequencing errors. Thus, in the present analysis, whenever feasible, we used pairs or triplets of (relatively) closely related species (e.g., mammals or nematodes) instead of a single species.

## Competing interests

The author(s) declare that they have no competing interests.

## Authors' contributions

IBR and EVK incepted the study and proposed the general principles of the tests for homoplasy. IBR, MC and KT implemented the tests and performed data analysis. MC and LC contributed to the statistical analysis of the results. IBR and EVK wrote the manuscript which was read, edited, and approved by all authors.

## Reviewers' comments

### Reviewer's report 1

Alex Kondrashov, University of Michigan, Ann Arbor, USA

This is an interesting paper. Studying homoplasy in some of the least homoplasy-prone traits associated with proteins is important, because only traits of proteins can be used to infer relationships between distant forms of life. Relating parallelism in protein evolution to Vavilov's homologous series also makes sense, partially. And a contribution to Coelomata-Ecdysozoa debate also makes sense – although I am not sure that this issue really wants to be resolved (what if the two successive branching events were separated by 10 My some 1000 Mya?).

**Author response: ***The last point is well appreciated, the Coelomata-Ecdysozoa conundrum, like other phylogenetic problems that date to "Big Bang" epochs, might not be particularly willing to give in. However, we view it as an interesting point in eukaryotic evolution for better understanding the potential and limitations of different phylogenetic methods*.

I do not have any hard criticisms – but here are some points worth considering:

1. Rare genomic changes (RGC) definitely are the way to go – as long as we have genome-scale data, so that we can observe enough of them. Indeed, homoplasy is the worst enemy of phylogenetic reconstructions, and RGCs are less prone to it.

2. I believe that talking about cladistics, synapomorphies, and Hennigian markers in the context of RGCs may be misleading. Cladistics or not, homoplasy-free traits are the best for phylogenetics (Darwin knew this) and if we can be sure that evolution was homoplasy-free, the most parsimonious tree is the correct one – see Felsenstein's book. This is true even if we do not know which states of RGCs are derived (rooting the tree is a separate problem) – the correct unrooted tree is still the maximally parsimonious one. And, in the simplest case of binary traits, it is very easy to see if the data are consistent with no homoplasy – this is the case as long as there are no pairwise conflicts between traits (a conflict between two traits appears if all 4 combinations of their states are present – see Pairwise Compatibility Theorem in Felsenstein's book).

**Author response: ***We believe that mentioning cladistics and derived characters will not mislead the reader, and this is, indeed, rather traditional in studies that involve RGCs. The reader interested in further insight will appreciate this comment*.

3. The most homoplasy-free RGCs are associated with large-scale events – deletions, insertions, and inversions (in particular, see Chaisson M. J., Raphael B. J., and Pevzner P. A. Microinversions in mammalian evolution. PNAS 2006 103: 19824–19829), because in this case multiple origins of the same state are unlikely. Substitutions (nucleotide or even amino acid) are not as good, because the number of possible trait states (4 or 20) is small enough to allow substantial homoplasy. Still, the kinds of substitutions used by the authors (RGC-CAMs, 2- or 3-nucleotide amino acid substitutions at conservative sites) are the closest thing to RGCs among all substitutions. Using them makes sense, due to two reasons. First, changes associated with non-coding sequences are not suitable for comparison of distant organisms, where such sequences are too dissimilar. Second, indels in conservative regions of proteins are probably too rare, even at the level of the whole proteome.

4. What about convergent evolution – because for an RGC-CAM there are more than two possible trait states, convergence is a possibility.

Author response

*It is hard to study convergent changes for the current dataset because the tree density *(Figure 1) *is insufficient. Some convergent changes might be counted as parallel changes*.

5. The point about Vavilov's homologous series is well-taken. However, parallel evolution can only explain a part of this phenomenon. Even more importantly, similar (not necessarily homologous; see Wooding S. et al. Independent evolution of bitter-taste sensitivity in humans and chimpanzees. Nature 440, 930 – 934, 2006) mutations and polymorphisms cause similar ("homologous") phenotypes in closely related species (this is discussed in Mednikov's book; Mednikov BM. The law of homologous series. To the 60^th ^anniversary of the discovery of the law by N. I. Vavilov. Moscow, Znanie, 1980). This second factor is probably the key reason for homologous variation within closely related species.

**Author response: ***It is suggested here that the parallel phenotypic changes observed by Vavilov could be caused by different mutations in orthologous genes. One should note, however, that in the exciting paper of Wooding et al., the mutations involved differentially impair the gene in question. This, indeed, could be rather widespread and could cause parallel emergence of some common phenotypes. However, the explanation of the higher prevalence of parallel mutations in closely related species steeped in the covarion model, as suggested here, applies to all kinds of mutations not just those that impair function, and in that regard, appears to be considerably more general*.

### Reviewer's report 2

Nicolas Galtier, CNRS, Université Montpellier 2, France

This manuscript develops on the observation that rare genomic changes (here, amino-acid substitutions observed only in the ingroup, not in outgroups, and requiring two or three nucleotide changes), which were selected for their supposed very small probability of appearing twice independently during evolution, actually reveal significant homoplasy, so that the branching order between arthropods, nematodes and vertebrates is still unresolved. Here Rogozin et al. ask what kinds of multiple change events have affected the evolution of these characters, and in which lineages they have occurred. Having detected more homoplasy in old than in recent branches, the authors invoke the covarion process as the probable reason for this unexpected pattern. I like this proposal, which I think makes sense, and for which I wish we get further support in the future. The authors finally argue that, correcting for these instances of homoplasy, the Coelomata hypothesis (nematodes, (arthropodes, deuterostomes)) is best supported.

I welcome publication of this manuscript in Biology Direct, especially since it belongs to a series of papers analysing these data – I hope that the discussion system offered by Biology Direct will enable a grouping of the arguments relevant to the (important) Coelomata/Ecdysozoa debate in a single editorial place, for the readers' benefit.

Now technical comments about the paper:

#### 1. Outgroup choice

In this study, all the possible combinations of eight outgroups are tried, and the average patterns are reported. Combinations of outgroups are given equal a priori weights, and unclear a posteriori weights – parallelism or reversal events supported by several choices of outgroup are counted once, so that combinations yielding similar results will be underweighted, as compared to combinations supporting unique events. Yet it is clear that the various outgroups do not have the same value: some are much closer to the ingroup than others, and therefore more reliable. Playing with outgroups significantly modifies the picture as far as the Coelomata/Ecdysozoa controversy is concerned, as noted by Irimia & Roy. If, for instance, one focuses on the 6th line of additional table 1, in which only the closest two outgroups (basal metazoa Trichoplax and Nematostella) are included, it appears that the (roughly estimated) expected number of nematode/insect parallel changes (5.8) is substantially less than the number of RGC_CAMs supporting Ecdysozoa (18, vs 7 for Coelomata), in contrast with the average (over outgroup combinations) pattern.

***Author response: ****Using only one set of outgroup species (e.g. in the cited paper of Irimia et al.) might be misleading because the numbers of shared RGC_CAMs are small and could be severely affected by statistical errors. Thus, the reviewer's estimate of the number parallel changes should be taken with a lot of caution. This being said, we cannot rule out the possibility that some of the apparent phylogenetic signal in support of Ecdysozoa is "real", i.e., due to a whole genome duplication and subsequent gene loss *(Ref. [14]). *See also our response to a similar comment of reviewer #3*.

#### 2. Focusing on internal branches

Parallel changes and reversals are mostly investigated in internal branches. Only once do the authors examine the Caenorhabditis/Mosquito pair of terminal branches for control. I am not sure why. Under the interesting hypothesis introduced in discussion, which states that parallel changes are more probable in closely related lineages thanks to a higher overlap between their sets of variable sites (covarions), one would expect a high rate of paralellism in the Apis/Aedes and Apis/Anopheles pairs of terminal branches, for instance.

**Author response: ***The reviewer suggests an analysis of three patterns:*

Am_*In*: Am = Y, Dm = X, Ag = Y, Aa = Y, all other species = X.

Am_Ag: Am = Y, Dm = X, Ag = Y, Aa = X, all other species = X.

Am_Aa: Am = Y, Dm = X, Ag = X, Aa = Y, all other species = X.

It is proposed that parallel changes in the branch leading to Am and the other insect branch could be a plausible explanation for these patterns. We analyzed these patterns:

**Table:** 

	Branches where parallel changes were analyzed		
	
	Am_*In*	Am_Ag	Am_Aa
#RGC_CAMs (%)	2 (25)	4 (50)	2 (25)
Relative branch length	0.211	0.457	0.332
Normalized #parallel changes	9.5	8.6	6.0

#RGC_CAs (%)	62 (54)	37 (33)	15 (13)
Relative branch length	0.258	0.428	0.315
Normalized #parallel changes	240.3	86.4	47.6

*The numbers of RGC_CAMs are small and so hard to interpret, however RGC_CAs, indeed, strongly supported the high rate of parallel changes within the insect clade, just as suggested by the reviewer. The raw number of RGC_CAs for Am_In (62) is 4-fold greater than that for In_ Dn *([16], see Table 1) *although the branch lengths are almost identical *(Figure 1*and results not shown). This is consistent with the prediction that parallel changes are more likely to occur in closely related lineages. However, there is also a potential problem with these analyses as there are alternative scenarios that could explain the observed patterns. For instance, the pattern Am_In can be explained by one X->Y mutation in the branch leading from the analyzed trifurcation to insects and another Y->X mutation in the branch leading to Dm. We cannot estimate the impact of such alternative scenarios at this stage*.

#### 3. Using maximum parsimony (MP)

I found a bit self-contradictory to use MP to unravel the history of these characters, when the premise of the study is that homoplasy is substantial – i.e., MP is mislead. If homoplasy is strong then the true scenario can be different from the MP scenario, which complicates the task of quantifying, and locating in the tree, events of reversal or parallelism. Specifically, I wonder whether errors of the MP method might contribute to the amazing pattern reported in this study – a higher amount of homoplasy in old branches.

Let me take the example of parallel changes. Parallel changes are invoked when (i) two distinct lineages, say A and B, share derived state Y, while other lineages carry the outgroup state X, and (ii) A and B are separated by at least two internal branches in the tree. This is so because if A and B are separated by a single internal branch (e.g. Apis and Drosophila) then the pattern can as well be explained by a reversal in the lineage branching in between (mosquitos). This is strict MP rationale. Now assume that evolution is not strictly parsimonious, and give a non-zero probability to suboptimal scenarios. If A and B are separated by exactly two internal branches, then a scenario with two reversals (in the two lineages branching between A and B) requires just one more step than the MP scenario. So you expect that a certain fraction of such characters will be falsely interpreted as old parallelisms, when they actually correspond to young reversals. The probability that multiple reversals induce a parallelism-like pattern decreases as the topological distance between A and B increases (since the number of required reversals increases), mimicking the covarion effect.

Here is how this could translate as far as the metazoa data set is concerned. Assume for a moment that (deuterostome, (nematode, insect)) is the true tree (Ecdysozoa hypothesis). Patterns supporting a N-Io parallel change could be explained by one X->Y change in the Ecdysozoa ancestor, and two Y->X reversals: one in Apis, one in Brugia. Patterns supporting a N-In parallel change would require three reversals (in Apis, Drosophila, and Brugia). This might partly explain why the latter are less frequently observed than the former.

MP can be mislead in many ways, and I do not mean to argue that non-parsimonious evolution will always result in older reconstructed than real changes – sometimes you can miss old changes as well. I suggest, however, that some caution is needed when interpreting these numbers – they should not be taken as errorless observations, and a careful study of the potential biases would be welcome. I note that these potential biases might propagate to further steps of the analysis, when MP-estimated branch lengths are used to roughly quantify the expected patterns in nematode, insect and deuterostome stem branches.

**Author response: ***Any phylogenetic method can mislead. We do not think that RGC_CAMs are different in this respect from other methods. However, taking into account that RGC_CAMs are rare events, we do not expect that non-parsimonious evolution will be as frequent as for all possible amino acid substitutions (see also the new *Ref. [43]). *We agree that interpretation of RGC_CAMs requires some caution in view of the use of parsimony and added the following to the text:*

"*Of course, interpretation of these results requires caution as with any result obtained with the parsimony principle. We inferred parallel changes and/or reversals under evolutionary scenarios that, according to maximum parsimony, required two substitutions *(Figures 3 and 6). *We cannot rule out that non-parsimonious scenarios that involve more than two parallel changes and/or reversals might have some impact on the RGC_CAs and RGC_CAMs analyses. However, it is expected that RGC_CAMs (and, to a lesser extent, RGC_CAs) are, largely, refractory to this problem because the probability of three independent rare events is much smaller than two independent rare events*."

#### 4. Taxon sampling

Generally, I have the feeling that taxon sampling is limiting in current study of rare genomic changes in metazoa. Our collective experience in other groups/time scales is that methodological debates lasted until a data set of reasonable size (taxonomy-wise) was available, after which a consensus arose. This was the case for mammals, for instance. I would bet that the addition of another ten genomes to this data set will resolve the main issues, both topological (Ecdysozoa or Coelomata?) and mechanistic (covarion-dependent homoplasy?).

**Author response: ***In our opinion, the decisive argument strongly supporting the rodent-primate clade was produced by Thomas et al. (Thomas JW, Touchman JW, Blakesley RW, et al. (71 co-authors). 2003. Comparative analyses of multi-species sequences from targeted genomic regions. Nature. 424: 788–793) using truly irreversible RGCs (insertions of mobile elements) for a relatively small number of species. In general, the number of species is not important for truly irreversible RGCs. Unfortunately, such RGCs are not available for distantly related species. Thus, unless the newly sequenced genomes help to resolve the issue of dramatic differences in evolutionary rates between nematodes, insects and mammals *(Figure 1), *we do not expect that adding a few more genomes will really help to resolve the issue*.

### Reviewer's report 3

Robert Lanfear and Maximilian J. Telford, University College London, London, UK (nominated by Laurence Hurst)

This is a very interesting and provocative paper. It has two main messages:

First, that careful consideration of their RGC-CAM database seems to support the Coelomata hypothesis ((arthropods, vertebrates) nematodes) over the broadly accepted Ecdysozoa hypothesis ((arthropods, nematodes) vertebrates).

Second, that the numbers of parallel changes in sister taxa are much more frequent close to their divergence point than later on, long after their divergence. This is proposed to be due to the effects of a covarion model which suggests similar genes (in similar genetic backgrounds?) will be constrained to undergo similar substitutions and this will be less true for more dissimilar genes.

We have a number of points that we feel need addressing.

As prompted by the Irimia analysis, the authors have gone to some pains to include a diversity of outgroups. They analyse the effects of all possible combinations of these outgroups and tally the number of times their RGC-CAMs support Coelomata and Ecdysozoa. Their conclusion is that there is very little difference in the support for Ceolomata and for Ecdysozoa.

However, we know that some outgroups (close ones) are expected *a priori *to be superior to more distant ones. From their additional file 1, we have extracted the experiments which contain 1 or both close outgroups (i.e. the metazoans *Trichoplax *and *Nematostella*) and compared these to those experiments with no metazoan outgroups. In the optimal cases (both metazoan outgroups included) 34 of 64 experiments support Ecdysozoa and 0 support Coelomata. Overall, including at least one metaozoan outgroup 88 analyses (out of 192) support Ecdysozoa and 22 support Coelomata. Support for Ecdysozoa is lower for *Trichoplax *only cases and higher for *Nematostella *only cases. In the worst cases (neither metazoan present in the analysis) ALL analyses support Coelomata. To us this suggests, that, at least in the absence of any corrections for branch lengths, their dataset supports Ecdysozoa.

Author response

*1) Correcting for branch lengths is absolutely crucial. This is addressed in some detail in *Ref. [21].

*2) The reviewers believe that Nematostella a priori is a superior outgroup. However, the internal branch between Nematostella and the analyzed trifurcation is extremely short (0 RGC_CAMs, *Figure 1), *so Nematostella is too close to the trifurcation to be a reliable outgroup. For instance, if there was a whole genome duplication and differential gene losses, Nematostella becomes an inappropriate outgroup that does not allow correct polarization RGC_CAMs*.

The results supporting the covarion model are fascinating and very exciting. We feel that this idea might be better tested in a dataset that has a less controversial phylogeny and with good constraints on branch lengths/divergence times. In particular, it seems likely that the often very small branch lengths estimated using the RGC_CAM approach could be particularly prone to error. Perhaps what we really mean is that the evidence from the current analysis needs further testing with a data set with fewer free parameters.

**Author response: ***We certainly appreciate this point and very well may return to the issue with other data sets. In the meantime, we added more analysis of parallel changes within the insect clade in our response to reviewer #2; the conclusions are strongly reinforced*.

Previously, the authors have used statistical tests to compare support for the Coelomata and Ecdysozoa hypotheses. These statistical tests were based on the assumption that "RGC_CAMs within a gene evolve independently of each other" (Ref. [14]). We are concerned that if the current paper is correct, and the covarion model applies, then this assumption does not hold. If this is the case, then previous tests between hypotheses might be invalid. Could the authors include some discussion of this point?

**Author response: ***First, it seems that, should the existence of covarions violate the independence assumption, this will affect any statistical models and tests used in phylogenetic analysis not only those employed in our previous RGC_CAM studies. Second, it is far from being obvious that, if the covarion model applies, the site independence assumption does not. There is no reason to believe that replacements within a covarion set existing at given time are not independent (although no replacements are allowed outside the covarion set), and since the covarion set changes fast during evolution, running through the protein sequence, the overall site independence seems a reasonable assumption. Of course, rigorous test of the covarion model and its effects on site independence are desirable, and it is our hope that this paper might attract some additional attention to the study of covarions that is currently not as popular as the subject, perhaps, deserves*.

"The RGC_CAM analysis has been combined with several statistical tests of competing phylogenetic hypotheses and has been shown to be robust to branch length differences and taxon sampling within a broad range of variation [14]"

We would argue that recent studies show that the RGC_CAM approach is actually very sensitive to taxon sampling – in both a recent critique of the approach and the current authors response to that critique, taxon sampling was shown to be a fundamental problem, with different taxon sets giving significantly different answers to the Coelomata/Ecdysozoa debate. Our re-analysis of the current dataset also supports the view that taxon sampling is of fundamental importance to the conclusions reached using this approach.

**Author response: ***We quite agree with the reviewers regarding the importance of taxon sampling. Therefore, here and in *Ref. [21], *we used all combinations from a large set of outgroup species. We believe this helps obtaining reliable results*.

Page 6:

"Moreover, the ecdysozoan topology is currently favored in the evo-devo community, on the basis of the perceived deep commonalities in the developmental processes of various molting animals [30,31]"

This is a misrepresentation of the evodevo community. There is really no 'perceived deep commonality' in the developmental basis of moulting – it is simply the case that moulting is the only thing that appears to unite the ecdysozoan animals, and as such it is an inferred rather than perceived commonality.

**Author response: ***If some of the leaders of the evo-devo community say so...corrected just as suggested*.

Page 7:

"However, as shown previously, when the branch lengths are taken into account, the support for the coelomate clade becomes substantially greater than that for Ecdysozoa [21]."

Can this be clarified please. It is not clear whether the authors corrected for branch lengths in the present analysis, or whether they are merely referring to their previous analysis in which they did correct for branch lengths (but used a rather more limited dataset).

**Author response: ***We referred to our previous results *(Ref. [21]).

If the authors haven't corrected for branch length in the current analysis, why not?

Finally, if the authors have corrected for branch length, can the above sentence be clarified to indicate the number of tests in which the Coelomata hypothesis receives significant support ('substantially greater' is a bit ambiguous – does it have anything to do with significance?), and the number in which Ecdysozoa receive significant support.

**Author response: ***In the above sentence, we refer to the previous results where the meaning of "substantial" is explicit. The main purpose of the present paper was not to provide additional arguments in the Coelomata-Ecdysozoa debate but to estimate the extent of parallel changes and reversals. Therefore, we did not give the results of statistical test in the text. However, since this seems to be of interest, here they are. After the essential correction for branch lengths, for 236 (93%) combinations of species, there was statistical support (Fisher's exact test) for the coelomate clade, whereas with the rest of the combinations (7%), none of the topologies received statistical support. This is very similar to our previous results *(Ref. [21]), *and we believe that this constitutes substantial support*.

## Supplementary Material

Additional file 1 Results of RGC_CAM analysis and estimates of homoplasy with sampling of outgroup species. Click here for file

Additional file 2 Frequency of amino acid substitutions. Click here for file

Additional file 3 Statistics of the comparisons of amino acid changes. Click here for file

Additional file 4 Time estimates of the nematodes-insects-vertebrates divergence. Click here for file
