# Supplementary material for: Homoplasy in genome-wide analysis of rare amino acid replacements: the molecular-evolutionary basis for Vavilov's law of homologous series
Source: Biol Direct. 2008 Mar 17;3:7. doi: 10.1186/1745-6150-3-7 (PMC2292158; doi:10.1186/1745-6150-3-7)
Supplement: Additional file 1 — Results of RGC_CAM analysis and estimates of homoplasy with sampling of outgroup species. [file 1745-6150-3-7-S1.doc]

Rogozin et al.

Additional file 1. Results of RGC_CAM analysis and estimates of homoplasy with sampling of outgroup species.

-------------------------------------------------------------------------------------------------------------------------------

Combination of Hypothesis Branch length Reversals Parallel changes

outgroup species C E B D I N Stem *N Io In Do Dn* *N_Io N_In N_Do N_Dn Io_Do In_Do Io_Dn In_Dn* *Pe*

(At,Sc,Sp,Pf

Cn,Ta,Nv,Dd)

-------------------------------------------------------------------------------------------------------------------------------

00000001 40 6 5 12 33 204 1276 24 4 6 5 2 4 0 0 1 1 0 1 0 5.46

00000010 12 23 3 7 44 341 188 5 1 0 7 5 5 1 0 1 0 0 0 0 5.70

00000011 3 4 1 3 29 188 15 1 1 0 1 0 4 0 0 1 0 0 0 0 4.90

00000100 22 24 2 6 42 313 258 5 3 1 5 0 6 1 0 1 0 0 1 0 5.69

00000101 5 5 0 3 27 171 23 1 1 1 1 0 4 0 0 1 0 0 1 0 5.18

00000110 7 18 0 3 39 284 22 1 1 0 3 0 5 1 0 1 0 0 0 0 5.79

00000111 2 4 0 2 25 158 3 1 1 0 0 0 4 0 0 1 0 0 0 0 4.85

00001000 60 13 7 9 27 195 1473 21 4 2 4 6 5 1 0 0 1 0 1 0 4.88

00001001 19 3 1 6 18 135 167 7 1 1 2 1 3 0 0 0 1 0 1 0 5.09

00001010 4 10 1 3 24 179 27 1 1 0 2 1 4 1 0 0 0 0 0 0 4.68

00001011 1 3 1 2 15 126 8 1 1 0 0 0 3 0 0 0 0 0 0 0 4.32

00001100 7 10 0 2 22 169 32 2 2 0 2 0 5 1 0 0 0 0 1 0 4.06

00001101 3 3 0 1 13 118 11 1 1 0 1 0 3 0 0 0 0 0 1 0 3.48

00001110 4 8 0 1 20 156 12 1 1 0 1 0 4 1 0 0 0 0 0 0 4.02

00001111 1 3 0 1 11 110 2 1 1 0 0 0 3 0 0 0 0 0 0 0 2.97

00010000 43 8 8 9 17 139 2610 31 5 2 5 5 3 0 0 0 0 0 1 0 4.21

00010001 13 3 0 5 10 104 228 4 2 1 2 0 2 0 0 0 0 0 1 0 2.80

00010010 2 4 0 1 13 127 20 1 1 0 1 1 3 0 0 0 0 0 0 0 3.25

00010011 1 1 0 1 8 99 7 1 1 0 0 0 2 0 0 0 0 0 0 0 2.36

00010100 2 6 0 2 13 117 26 1 1 0 2 0 3 0 0 0 0 0 1 0 3.17

00010101 2 2 0 2 7 88 9 1 1 0 1 0 2 0 0 0 0 0 1 0 1.93

00010110 1 3 0 1 11 110 5 1 1 0 1 0 3 0 0 0 0 0 0 0 2.81

00010111 1 1 0 1 6 84 2 1 1 0 0 0 2 0 0 0 0 0 0 0 1.76

00011000 15 2 3 4 12 86 207 5 1 1 1 1 3 0 0 0 0 0 1 0 4.17

00011001 9 1 0 3 9 76 69 2 1 1 1 0 2 0 0 0 0 0 1 0 4.02

00011010 1 1 0 1 10 83 10 1 1 0 0 0 3 0 0 0 0 0 0 0 3.77

00011011 1 1 0 1 7 74 6 1 1 0 0 0 2 0 0 0 0 0 0 0 3.44

00011100 2 2 0 1 9 75 13 1 1 0 1 0 3 0 0 0 0 0 1 0 2.96

00011101 2 1 0 1 6 67 7 1 1 0 1 0 2 0 0 0 0 0 1 0 2.59

00011110 1 1 0 1 8 73 5 1 1 0 0 0 3 0 0 0 0 0 0 0 2.88

00011111 1 1 0 1 5 65 2 1 1 0 0 0 2 0 0 0 0 0 0 0 2.37

00100000 51 13 7 12 24 205 1419 21 5 1 3 7 4 1 0 0 1 0 1 0 4.27

00100001 15 4 0 6 14 139 172 4 2 0 2 1 3 0 0 0 1 0 1 0 3.14

00100010 7 9 0 2 21 180 25 1 1 0 1 1 4 1 0 0 0 0 0 0 4.09

00100011 2 3 0 1 12 126 8 1 1 0 0 0 3 0 0 0 0 0 0 0 2.70

00100100 11 11 0 2 21 176 39 1 1 0 1 0 4 1 0 0 0 0 1 0 4.16

00100101 5 3 0 2 11 122 13 1 1 0 1 0 3 0 0 0 0 0 1 0 2.81

00100110 6 8 0 1 19 161 10 1 1 0 0 0 4 1 0 0 0 0 0 0 4.35

00100111 2 3 0 1 10 112 3 1 1 0 0 0 3 0 0 0 0 0 0 0 2.58

00101000 27 8 4 6 17 149 392 8 1 0 3 3 3 1 0 0 1 0 1 0 4.00

00101001 11 3 0 4 10 111 95 2 1 0 2 1 2 0 0 0 1 0 1 0 3.30

00101010 4 6 0 2 15 138 13 1 1 0 1 0 3 1 0 0 0 0 0 0 3.93

00101011 1 3 0 1 8 104 5 1 1 0 0 0 2 0 0 0 0 0 0 0 2.71

00101100 6 8 0 1 14 134 18 1 1 0 1 0 3 1 0 0 0 0 1 0 3.37

00101101 3 3 0 1 7 101 8 1 1 0 1 0 2 0 0 0 0 0 1 0 2.27

00101110 4 6 0 1 13 126 7 1 1 0 0 0 3 1 0 0 0 0 0 0 3.57

00101111 1 3 0 1 6 95 2 1 1 0 0 0 2 0 0 0 0 0 0 0 2.00

00110000 13 6 3 4 12 96 212 4 2 0 1 2 3 0 0 0 0 0 1 0 3.98

00110001 8 2 0 3 7 78 72 2 2 0 1 0 2 0 0 0 0 0 1 0 2.46

00110010 1 3 0 1 9 89 5 1 1 0 0 1 3 0 0 0 0 0 0 0 3.07

00110011 1 1 0 1 5 74 4 1 1 0 0 0 2 0 0 0 0 0 0 0 1.87

00110100 2 5 0 2 10 84 13 1 1 0 1 0 3 0 0 0 0 0 1 0 3.46

00110101 2 1 0 2 5 68 7 1 1 0 1 0 2 0 0 0 0 0 1 0 1.85

00110110 1 3 0 1 8 80 3 1 1 0 0 0 3 0 0 0 0 0 0 0 2.95

00110111 1 1 0 1 4 65 2 1 1 0 0 0 2 0 0 0 0 0 0 0 1.60

00111000 12 2 2 3 9 75 115 4 1 0 1 0 3 0 0 0 0 0 1 0 3.51

00111001 8 1 0 2 6 66 48 2 1 0 1 0 2 0 0 0 0 0 1 0 2.83

00111010 1 1 0 1 7 72 5 1 1 0 0 0 3 0 0 0 0 0 0 0 2.92

00111011 1 1 0 1 4 64 4 1 1 0 0 0 2 0 0 0 0 0 0 0 2.07

00111100 2 2 0 1 7 67 8 1 1 0 1 0 3 0 0 0 0 0 1 0 2.66

00111101 2 1 0 1 4 59 5 1 1 0 1 0 2 0 0 0 0 0 1 0 1.85

00111110 1 1 0 1 6 65 3 1 1 0 0 0 3 0 0 0 0 0 0 0 2.48

00111111 1 1 0 1 3 57 2 1 1 0 0 0 2 0 0 0 0 0 0 0 1.52

01000000 57 13 7 12 21 186 1928 19 2 3 3 4 2 1 0 1 1 0 1 0 4.52

01000001 14 3 0 5 15 121 172 4 1 0 2 1 2 0 0 0 1 0 1 0 4.38

01000010 6 10 0 3 20 169 20 0 0 0 1 0 2 1 0 0 0 0 0 0 4.58

01000011 2 3 0 2 14 112 5 0 0 0 0 0 2 0 0 0 0 0 0 0 4.19

01000100 10 10 0 1 17 157 36 0 0 0 1 0 2 1 0 0 0 0 1 0 3.90

01000101 4 3 0 1 11 104 12 0 0 0 1 0 2 0 0 0 0 0 1 0 3.28

01000110 5 7 0 1 17 145 8 0 0 0 0 0 2 1 0 0 0 0 0 0 4.00

01000111 2 3 0 1 11 97 1 0 0 0 0 0 2 0 0 0 0 0 0 0 3.38

01001000 27 9 3 6 16 134 393 6 0 0 3 3 1 1 0 0 1 0 1 0 4.02

01001001 10 3 0 4 11 95 89 2 0 0 2 1 1 0 0 0 1 0 1 0 3.37

01001010 3 7 0 2 15 126 14 0 0 0 1 0 1 1 0 0 0 0 0 0 3.87

01001011 1 3 0 1 10 90 4 0 0 0 0 0 1 0 0 0 0 0 0 0 3.17

01001100 5 8 0 0 12 120 17 0 0 0 1 0 1 1 0 0 0 0 1 0 3.08

01001101 2 3 0 0 7 85 8 0 0 0 1 0 1 0 0 0 0 0 1 0 2.09

01001110 3 6 0 0 12 113 8 0 0 0 0 0 1 1 0 0 0 0 0 0 3.19

01001111 1 3 0 0 7 81 1 0 0 0 0 0 1 0 0 0 0 0 0 0 2.18

01010000 13 4 4 3 10 86 237 6 0 1 1 0 1 0 0 0 0 0 1 0 4.10

01010001 8 1 0 1 7 68 74 1 0 0 1 0 1 0 0 0 0 0 1 0 3.40

01010010 1 3 0 0 9 82 5 0 0 0 0 0 1 0 0 0 0 0 0 0 4.01

01010011 1 1 0 0 6 65 3 0 0 0 0 0 1 0 0 0 0 0 0 0 3.15

01010100 2 3 0 0 8 76 12 0 0 0 1 0 1 0 0 0 0 0 1 0 3.12

01010101 2 1 0 0 5 59 7 0 0 0 1 0 1 0 0 0 0 0 1 0 2.31

01010110 1 2 0 0 8 73 3 0 0 0 0 0 1 0 0 0 0 0 0 0 3.43

01010111 1 1 0 0 5 57 1 0 0 0 0 0 1 0 0 0 0 0 0 0 2.53

01011000 10 2 3 2 8 62 114 2 0 0 1 0 1 0 0 0 0 0 1 0 3.01

01011001 7 1 0 1 6 55 51 1 0 0 1 0 1 0 0 0 0 0 1 0 2.69

01011010 1 1 0 0 7 61 5 0 0 0 0 0 1 0 0 0 0 0 0 0 2.94

01011011 1 1 0 0 5 54 3 0 0 0 0 0 1 0 0 0 0 0 0 0 2.50

01011100 2 2 0 0 6 56 9 0 0 0 1 0 1 0 0 0 0 0 1 0 2.22

01011101 2 1 0 0 4 49 5 0 0 0 1 0 1 0 0 0 0 0 1 0 1.78

01011110 1 1 0 0 6 55 3 0 0 0 0 0 1 0 0 0 0 0 0 0 2.49

01011111 1 1 0 0 4 48 1 0 0 0 0 0 1 0 0 0 0 0 0 0 1.98

01100000 27 9 3 7 17 146 467 7 0 0 3 4 2 1 0 0 1 0 1 0 4.65

01100001 11 3 0 3 11 104 104 2 0 0 2 1 2 0 0 0 1 0 1 0 3.54

01100010 5 7 0 1 16 133 12 0 0 0 1 0 2 1 0 0 0 0 0 0 4.41

01100011 2 3 0 0 10 95 5 0 0 0 0 0 2 0 0 0 0 0 0 0 3.25

01100100 8 8 0 0 15 130 22 0 0 0 1 0 2 1 0 0 0 0 1 0 4.53

01100101 4 3 0 0 9 93 10 0 0 0 1 0 2 0 0 0 0 0 1 0 3.12

01100110 4 6 0 0 15 120 6 0 0 0 0 0 2 1 0 0 0 0 0 0 4.57

01100111 2 3 0 0 9 86 1 0 0 0 0 0 2 0 0 0 0 0 0 0 3.18

01101000 19 7 2 5 13 115 248 3 0 0 3 3 1 1 0 0 1 0 1 0 3.78

01101001 8 3 0 3 8 86 73 1 0 0 2 1 1 0 0 0 1 0 1 0 2.73

01101010 3 5 0 1 12 108 10 0 0 0 1 0 1 1 0 0 0 0 0 0 3.59

01101011 1 3 0 0 7 81 4 0 0 0 0 0 1 0 0 0 0 0 0 0 2.48

01101100 4 7 0 0 11 105 13 0 0 0 1 0 1 1 0 0 0 0 1 0 3.38

01101101 2 3 0 0 6 78 7 0 0 0 1 0 1 0 0 0 0 0 1 0 2.05

01101110 3 5 0 0 11 99 6 0 0 0 0 0 1 1 0 0 0 0 0 0 3.51

01101111 1 3 0 0 6 74 1 0 0 0 0 0 1 0 0 0 0 0 0 0 2.15

01110000 10 3 3 2 8 71 127 2 0 0 1 0 1 0 0 0 0 0 1 0 3.69

01110001 7 1 0 1 5 59 55 1 0 0 1 0 1 0 0 0 0 0 1 0 2.41

01110010 1 2 0 0 7 68 4 0 0 0 0 0 1 0 0 0 0 0 0 0 3.46

01110011 1 1 0 0 4 56 3 0 0 0 0 0 1 0 0 0 0 0 0 0 2.07

01110100 2 3 0 0 7 64 9 0 0 0 1 0 1 0 0 0 0 0 1 0 3.19

01110101 2 1 0 0 4 52 6 0 0 0 1 0 1 0 0 0 0 0 1 0 1.89

01110110 1 2 0 0 7 62 2 0 0 0 0 0 1 0 0 0 0 0 0 0 3.47

01110111 1 1 0 0 4 50 1 0 0 0 0 0 1 0 0 0 0 0 0 0 2.07

01111000 10 2 2 2 6 56 89 2 0 0 1 0 1 0 0 0 0 0 1 0 2.44

01111001 7 1 0 1 4 49 42 1 0 0 1 0 1 0 0 0 0 0 1 0 1.82

01111010 1 1 0 0 5 55 4 0 0 0 0 0 1 0 0 0 0 0 0 0 2.24

01111011 1 1 0 0 3 48 3 0 0 0 0 0 1 0 0 0 0 0 0 0 1.52

01111100 2 2 0 0 5 51 6 0 0 0 1 0 1 0 0 0 0 0 1 0 2.06

01111101 2 1 0 0 3 44 4 0 0 0 1 0 1 0 0 0 0 0 1 0 1.39

01111110 1 1 0 0 5 50 2 0 0 0 0 0 1 0 0 0 0 0 0 0 2.27

01111111 1 1 0 0 3 43 1 0 0 0 0 0 1 0 0 0 0 0 0 0 1.55

10000000 47 16 3 8 29 205 1193 16 6 5 5 7 4 1 0 2 0 0 1 0 4.45

10000001 14 5 1 4 20 145 197 5 1 2 2 2 2 0 0 1 0 0 1 0 4.24

10000010 6 11 0 1 24 185 24 2 1 0 1 0 3 1 0 1 0 0 0 0 3.77

10000011 2 3 0 1 17 136 8 1 1 0 0 0 2 0 0 1 0 0 0 0 3.72

10000100 8 12 0 2 24 173 32 1 2 0 2 0 4 1 0 1 0 0 1 0 3.82

10000101 3 4 0 2 17 124 16 1 1 0 1 0 2 0 0 1 0 0 1 0 3.58

10000110 5 10 0 1 22 160 10 1 1 0 1 0 3 1 0 1 0 0 0 0 3.69

10000111 2 3 0 1 15 117 2 1 1 0 0 0 2 0 0 1 0 0 0 0 3.28

10001000 23 7 1 2 18 138 178 5 2 1 1 3 4 1 0 0 0 0 1 0 3.94

10001001 12 2 0 2 14 105 72 3 1 0 1 1 2 0 0 0 0 0 1 0 4.26

10001010 3 5 0 0 15 129 13 1 1 0 0 0 3 1 0 0 0 0 0 0 3.25

10001011 1 2 0 0 11 100 6 1 1 0 0 0 2 0 0 0 0 0 0 0 3.45

10001100 5 6 0 0 15 122 16 1 2 0 1 0 4 1 0 0 0 0 1 0 3.44

10001101 2 2 0 0 11 93 7 1 1 0 1 0 2 0 0 0 0 0 1 0 3.17

10001110 3 5 0 0 13 116 7 1 1 0 0 0 3 1 0 0 0 0 0 0 3.02

10001111 1 2 0 0 9 89 1 1 1 0 0 0 2 0 0 0 0 0 0 0 2.69

10010000 14 6 2 4 13 98 215 4 3 0 4 1 3 0 0 0 0 0 1 0 3.77

10010001 9 3 0 2 8 80 94 2 1 0 2 0 2 0 0 0 0 0 1 0 2.80

10010010 1 3 0 0 10 93 8 1 1 0 1 0 3 0 0 0 0 0 0 0 3.10

10010011 1 1 0 0 6 77 5 1 1 0 0 0 2 0 0 0 0 0 0 0 2.31

10010100 2 4 0 1 11 84 9 1 1 0 2 0 3 0 0 0 0 0 1 0 2.90

10010101 2 2 0 1 7 68 6 1 1 0 1 0 2 0 0 0 0 0 1 0 2.22

10010110 1 2 0 0 10 81 2 1 1 0 1 0 3 0 0 0 0 0 0 0 2.87

10010111 1 1 0 0 6 66 1 1 1 0 0 0 2 0 0 0 0 0 0 0 2.11

10011000 9 2 1 1 9 70 74 3 1 0 1 1 3 0 0 0 0 0 1 0 3.27

10011001 8 1 0 1 7 62 41 2 1 0 1 0 2 0 0 0 0 0 1 0 3.21

10011010 1 1 0 0 7 67 6 1 1 0 0 0 3 0 0 0 0 0 0 0 2.74

10011011 1 1 0 0 5 60 4 1 1 0 0 0 2 0 0 0 0 0 0 0 2.54

10011100 2 2 0 0 8 62 6 1 1 0 1 0 3 0 0 0 0 0 1 0 2.79

10011101 2 1 0 0 6 55 4 1 1 0 1 0 2 0 0 0 0 0 1 0 2.67

10011110 1 1 0 0 7 60 2 1 1 0 0 0 3 0 0 0 0 0 0 0 2.67

10011111 1 1 0 0 5 53 1 1 1 0 0 0 2 0 0 0 0 0 0 0 2.45

10100000 20 7 1 3 15 141 179 5 2 0 1 2 3 1 0 0 0 0 1 0 3.05

10100001 10 3 0 2 10 110 83 2 1 0 1 1 2 0 0 0 0 0 1 0 2.64

10100010 5 5 0 0 13 127 10 1 1 0 0 0 3 1 0 0 0 0 0 0 2.78

10100011 2 2 0 0 8 101 5 1 1 0 0 0 2 0 0 0 0 0 0 0 2.15

10100100 7 6 0 1 13 125 18 1 1 0 1 0 3 1 0 0 0 0 1 0 2.83

10100101 3 2 0 1 8 99 12 1 1 0 1 0 2 0 0 0 0 0 1 0 2.18

10100110 5 5 0 0 12 116 6 1 1 0 0 0 3 1 0 0 0 0 0 0 2.82

10100111 2 2 0 0 7 92 2 1 1 0 0 0 2 0 0 0 0 0 0 0 1.96

10101000 13 5 1 1 11 113 108 4 1 0 1 2 3 1 0 0 0 0 1 0 3.17

10101001 9 2 0 1 7 90 54 2 1 0 1 1 2 0 0 0 0 0 1 0 2.52

10101010 3 4 0 0 9 105 7 1 1 0 0 0 3 1 0 0 0 0 0 0 2.60

10101011 1 2 0 0 5 85 4 1 1 0 0 0 2 0 0 0 0 0 0 0 1.86

10101100 4 5 0 0 9 102 11 1 1 0 1 0 3 1 0 0 0 0 1 0 2.69

10101101 2 2 0 0 5 82 7 1 1 0 1 0 2 0 0 0 0 0 1 0 1.75

10101110 3 4 0 0 8 97 4 1 1 0 0 0 3 1 0 0 0 0 0 0 2.46

10101111 1 2 0 0 4 78 1 1 1 0 0 0 2 0 0 0 0 0 0 0 1.46

10110000 8 4 1 1 9 75 76 3 1 0 1 0 3 0 0 0 0 0 1 0 3.11

10110001 7 2 0 1 6 64 46 2 1 0 1 0 2 0 0 0 0 0 1 0 2.36

10110010 1 2 0 0 7 71 4 1 1 0 0 0 3 0 0 0 0 0 0 0 2.58

10110011 1 1 0 0 4 61 3 1 1 0 0 0 2 0 0 0 0 0 0 0 1.72

10110100 2 3 0 1 8 67 8 1 1 0 1 0 3 0 0 0 0 0 1 0 2.63

10110101 2 1 0 1 5 57 6 1 1 0 1 0 2 0 0 0 0 0 1 0 1.87

10110110 1 2 0 0 7 65 2 1 1 0 0 0 3 0 0 0 0 0 0 0 2.53

10110111 1 1 0 0 4 55 1 1 1 0 0 0 2 0 0 0 0 0 0 0 1.66

10111000 7 2 1 0 7 63 56 3 1 0 1 0 3 0 0 0 0 0 1 0 2.76

10111001 7 1 0 0 5 55 33 2 1 0 1 0 2 0 0 0 0 0 1 0 2.40

10111010 1 1 0 0 5 60 4 1 1 0 0 0 3 0 0 0 0 0 0 0 2.12

10111011 1 1 0 0 3 53 3 1 1 0 0 0 2 0 0 0 0 0 0 0 1.59

10111100 2 2 0 0 6 57 5 1 1 0 1 0 3 0 0 0 0 0 1 0 2.32

10111101 2 1 0 0 4 50 4 1 1 0 1 0 2 0 0 0 0 0 1 0 1.89

10111110 1 1 0 0 5 55 2 1 1 0 0 0 3 0 0 0 0 0 0 0 2.11

10111111 1 1 0 0 3 48 1 1 1 0 0 0 2 0 0 0 0 0 0 0 1.57

11000000 19 6 1 3 15 129 154 5 0 0 1 2 1 1 0 1 0 0 1 0 3.83

11000001 9 2 0 2 11 97 66 3 0 0 1 1 1 0 0 0 0 0 1 0 3.81

11000010 3 5 0 1 14 119 8 0 0 0 0 0 1 1 0 0 0 0 0 0 3.65

11000011 2 2 0 1 10 90 3 0 0 0 0 0 1 0 0 0 0 0 0 0 3.60

11000100 6 5 0 1 12 110 14 0 0 0 1 0 1 1 0 0 0 0 1 0 3.05

11000101 3 2 0 1 8 84 9 0 0 0 1 0 1 0 0 0 0 0 1 0 2.67

11000110 3 4 0 1 12 103 4 0 0 0 0 0 1 1 0 0 0 0 0 0 3.12

11000111 2 2 0 1 8 79 0 0 0 0 0 0 1 0 0 0 0 0 0 0 2.78

11001000 13 5 1 1 11 98 96 3 0 0 1 2 1 1 0 0 0 0 1 0 3.07

11001001 8 2 0 1 8 76 48 2 0 0 1 1 1 0 0 0 0 0 1 0 2.71

11001010 2 4 0 0 10 93 7 0 0 0 0 0 1 1 0 0 0 0 0 0 2.84

11001011 1 2 0 0 7 72 3 0 0 0 0 0 1 0 0 0 0 0 0 0 2.46

11001100 4 5 0 0 8 89 11 0 0 0 1 0 1 1 0 0 0 0 1 0 2.33

11001101 2 2 0 0 5 69 6 0 0 0 1 0 1 0 0 0 0 0 1 0 1.67

11001110 2 4 0 0 8 85 4 0 0 0 0 0 1 1 0 0 0 0 0 0 2.39

11001111 1 2 0 0 5 66 0 0 0 0 0 0 1 0 0 0 0 0 0 0 1.75

11010000 6 3 1 1 8 68 72 2 0 0 1 0 1 0 0 0 0 0 1 0 3.47

11010001 6 1 0 0 6 56 43 1 0 0 1 0 1 0 0 0 0 0 1 0 3.11

11010010 1 2 0 0 7 65 3 0 0 0 0 0 1 0 0 0 0 0 0 0 3.31

11010011 1 1 0 0 5 54 2 0 0 0 0 0 1 0 0 0 0 0 0 0 2.87

11010100 2 2 0 0 7 60 7 0 0 0 1 0 1 0 0 0 0 0 1 0 2.88

11010101 2 1 0 0 5 49 5 0 0 0 1 0 1 0 0 0 0 0 1 0 2.47

11010110 1 1 0 0 7 58 1 0 0 0 0 0 1 0 0 0 0 0 0 0 3.18

11010111 1 1 0 0 5 48 0 0 0 0 0 0 1 0 0 0 0 0 0 0 2.79

11011000 6 2 1 0 6 52 52 2 0 0 1 0 1 0 0 0 0 0 1 0 2.55

11011001 6 1 0 0 5 46 33 1 0 0 1 0 1 0 0 0 0 0 1 0 2.43

11011010 1 1 0 0 5 51 3 0 0 0 0 0 1 0 0 0 0 0 0 0 2.36

11011011 1 1 0 0 4 45 2 0 0 0 0 0 1 0 0 0 0 0 0 0 2.20

11011100 2 2 0 0 5 48 5 0 0 0 1 0 1 0 0 0 0 0 1 0 2.13

11011101 2 1 0 0 4 42 3 0 0 0 1 0 1 0 0 0 0 0 1 0 1.96

11011110 1 1 0 0 5 47 1 0 0 0 0 0 1 0 0 0 0 0 0 0 2.37

11011111 1 1 0 0 4 41 0 0 0 0 0 0 1 0 0 0 0 0 0 0 2.22

11100000 12 5 1 1 13 107 97 3 0 0 1 2 1 1 0 0 0 0 1 0 4.04

11100001 8 2 0 1 9 86 58 1 0 0 1 1 1 0 0 0 0 0 1 0 3.50

11100010 3 4 0 0 12 98 7 0 0 0 0 0 1 1 0 0 0 0 0 0 3.77

11100011 2 2 0 0 8 79 3 0 0 0 0 0 1 0 0 0 0 0 0 0 3.19

11100100 5 5 0 0 11 95 12 0 0 0 1 0 1 1 0 0 0 0 1 0 3.63

11100101 3 2 0 0 7 77 9 0 0 0 1 0 1 0 0 0 0 0 1 0 2.72

11100110 3 4 0 0 11 89 3 0 0 0 0 0 1 1 0 0 0 0 0 0 3.68

11100111 2 2 0 0 7 72 0 0 0 0 0 0 1 0 0 0 0 0 0 0 2.80

11101000 9 5 1 1 9 87 74 2 0 0 1 2 1 1 0 0 0 0 1 0 2.84

11101001 7 2 0 1 6 70 43 1 0 0 1 1 1 0 0 0 0 0 1 0 2.23

11101010 2 4 0 0 8 82 6 0 0 0 0 0 1 1 0 0 0 0 0 0 2.57

11101011 1 2 0 0 5 66 3 0 0 0 0 0 1 0 0 0 0 0 0 0 1.93

11101100 3 5 0 0 7 80 9 0 0 0 1 0 1 1 0 0 0 0 1 0 2.37

11101101 2 2 0 0 4 64 6 0 0 0 1 0 1 0 0 0 0 0 1 0 1.49

11101110 2 4 0 0 7 76 3 0 0 0 0 0 1 1 0 0 0 0 0 0 2.45

11101111 1 2 0 0 4 61 0 0 0 0 0 0 1 0 0 0 0 0 0 0 1.56

11110000 6 2 1 0 7 59 54 2 0 0 1 0 1 0 0 0 0 0 1 0 3.30

11110001 6 1 0 0 5 51 37 1 0 0 1 0 1 0 0 0 0 0 1 0 2.58

11110010 1 1 0 0 6 57 3 0 0 0 0 0 1 0 0 0 0 0 0 0 3.10

11110011 1 1 0 0 4 49 2 0 0 0 0 0 1 0 0 0 0 0 0 0 2.28

11110100 2 2 0 0 6 54 6 0 0 0 1 0 1 0 0 0 0 0 1 0 2.82

11110101 2 1 0 0 4 46 5 0 0 0 1 0 1 0 0 0 0 0 1 0 2.04

11110110 1 1 0 0 6 53 1 0 0 0 0 0 1 0 0 0 0 0 0 0 3.14

11110111 1 1 0 0 4 45 0 0 0 0 0 0 1 0 0 0 0 0 0 0 2.31

11111000 6 2 1 0 5 48 43 2 0 0 1 0 1 0 0 0 0 0 1 0 2.13

11111001 6 1 0 0 4 42 29 1 0 0 1 0 1 0 0 0 0 0 1 0 1.91

11111010 1 1 0 0 4 47 3 0 0 0 0 0 1 0 0 0 0 0 0 0 1.90

11111011 1 1 0 0 3 41 2 0 0 0 0 0 1 0 0 0 0 0 0 0 1.62

11111100 2 2 0 0 4 45 4 0 0 0 1 0 1 0 0 0 0 0 1 0 1.76

11111101 2 1 0 0 3 39 3 0 0 0 1 0 1 0 0 0 0 0 1 0 1.49

11111110 1 1 0 0 4 44 1 0 0 0 0 0 1 0 0 0 0 0 0 0 1.96

11111111 1 1 0 0 3 38 0 0 0 0 0 0 1 0 0 0 0 0 0 0 1.68

-------------------------------------------------------------------------------------------------------------------------------

N = the nematode internal branch; Io = the "old" (closer to the root) insect internal branch; In = the "new" (closer to the terminal branches) insect internal branch; Do = the "old" deuterostome internal branch; Dn = the "new" deuterostome internal branch.
