# Supplementary material for: Homoplasy in genome-wide analysis of rare amino acid replacements: the molecular-evolutionary basis for Vavilov's law of homologous series
Source: Biol Direct. 2008 Mar 17;3:7. doi: 10.1186/1745-6150-3-7 (PMC2292158; doi:10.1186/1745-6150-3-7)
Supplement: Additional file 2 — Frequency of amino acid substitutions. [file 1745-6150-3-7-S2.doc]

Rogozin et al.

##### Additional file 2: Frequency of amino acid substitutions.

Figure A. Histogram showing mutated amino acid across deuterosomes (D), insects (I) and nematodes (N). C_aa denotes the frequency of amino acids conserved in all 19 species.

Figure B. Histogram showing resulting amino acid substitutions across deuterosomes (D), insects (I) and nematodes (N).
