# Supplementary material for: Homoplasy in genome-wide analysis of rare amino acid replacements: the molecular-evolutionary basis for Vavilov's law of homologous series
Source: Biol Direct. 2008 Mar 17;3:7. doi: 10.1186/1745-6150-3-7 (PMC2292158; doi:10.1186/1745-6150-3-7)
Supplement: Additional file 3 — Statistics of the comparisons of amino acid changes. [file 1745-6150-3-7-S3.doc]

Rogozin et al.

Additional file 3

Statistics of the comparisons of amino acid changes in insects, nematodes and deuterostomes using two different tests, χ^2^ and linear correlation coefficient (CC).

|  | Mutated amino acids | | | Resulting amino acids | | |
| --- | --- | --- | --- | --- | --- | --- |
|  | Insects | Nematodes | Deuterostomes | Insects | Nematodes | Deuterostomes |
| Insects |  | χ^2^=16.6  P > 0.05 | χ^2^=27.6  P > 0.05 |  | χ^2^=21.2  P > 0.05 | χ^2^=21.9  P > 0.05 |
| Nematodes | CC=0.57 P < 0.01 |  | χ^2^= 4.6  P > 0.05 | CC=0.51 P < 0.05 |  | χ^2^=11.9  P > 0.05 |
| Deuterostomes | CC=0.27  P > 0.05 | CC=0.27  P > 0.05 |  | CC=0.41  P > 0.05 | CC=0.77 P < 0.01 |  |
