# Supplementary material for: Homoplasy in genome-wide analysis of rare amino acid replacements: the molecular-evolutionary basis for Vavilov's law of homologous series
Source: Biol Direct. 2008 Mar 17;3:7. doi: 10.1186/1745-6150-3-7 (PMC2292158; doi:10.1186/1745-6150-3-7)
Supplement: Additional file 4 — Time estimates of the nematodes-insects-vertebrates divergence. [file 1745-6150-3-7-S4.doc]

Rogozin et al.

Additional file 4

Time estimates of the nematodes-insects-vertebrates divergence.

| Species | L_1_ | L_2_ | L_3_ | T_e_ (Mya) |
| --- | --- | --- | --- | --- |
| Human | 1 | 7 | 1 | 855 |
| Mouse | 1 | 7 | 3 | 348 |
| *A. aegypti* | 8 | 6 | 10 | 568 |
| *A. gambiae* | 8 | 6 | 11 | 600 |
| *C. elegans* | 38 | 34 | 11 | 603 |
| *C. briggsae* | 38 | 34 | 7 | 902 |

L1 and L2 are internal branch lengths. L3 is the terminal branch length (see Figure 1 for details).
